# Newly identified Gon4l/Udu-interacting proteins implicate novel functions

**DOI:** 10.1038/s41598-020-70855-9

**Published:** 2020-08-26

**Authors:** Su-Mei Tsai, Kuo-Chang Chu, Yun-Jin Jiang

**Affiliations:** 1 Institute of Molecular and Genomic Medicine, National Health Research Institutes, Zhunan, Miaoli County Taiwan; 2 Laboratory of Developmental Signalling and Patterning, Institute of Molecular and Cell Biology, Singapore, Singapore; 3 Biotechnology Center, National Chung Hsing University, Taichung, Taiwan; 4 Institute of Molecular and Cellular Biology, National Taiwan University, Taipei, Taiwan; 5 Department of Life Science, Tunghai University, Taichung, Taiwan

**Keywords:** Biochemistry, Biological techniques, Developmental biology, Molecular biology

## Abstract

Mutations of the *Gon4l*/*udu* gene in different organisms give rise to diverse phenotypes. Although the effects of Gon4l/Udu in transcriptional regulation have been demonstrated, they cannot solely explain the observed characteristics among species. To further understand the function of Gon4l/Udu, we used yeast two-hybrid (Y2H) screening to identify interacting proteins in zebrafish and mouse systems, confirmed the interactions by co-immunoprecipitation assay, and found four novel Gon4l-interacting proteins: BRCA1 associated protein-1 (Bap1), DNA methyltransferase 1 (Dnmt1), Tho complex 1 (Thoc1, also known as Tho1 or HPR1), and Cryptochrome circadian regulator 3a (Cry3a). Furthermore, all known Gon4l/Udu-interacting proteins—as found in this study, in previous reports, and in online resources—were investigated by Phenotype Enrichment Analysis. The most enriched phenotypes identified include increased embryonic tissue cell apoptosis, embryonic lethality, increased T cell derived lymphoma incidence, decreased cell proliferation, chromosome instability, and abnormal dopamine level, characteristics that largely resemble those observed in reported *Gon4l*/*udu* mutant animals. Similar to the expression pattern of *udu*, those of *bap1*, *dnmt1*, *thoc1*, and *cry3a* are also found in the brain region and other tissues. Thus, these findings indicate novel mechanisms of Gon4l/Udu in regulating CpG methylation, histone expression/modification, DNA repair/genomic stability, and RNA binding/processing/export.

## Introduction

Gon4l is a nuclear protein conserved among species. Animal models from invertebrates to vertebrates have shown that the protein Gon4-like (Gon4l) is essential for regulating cell proliferation and differentiation. For example, the *Gon4l* homologous gene in *Caenorhabditis elegans*, *gon-4*, is critical for gonadogenesis. In *gon-4* mutant nematodes, precursor cells for somatic gonadal tissues are unable to divide or cell division is severely delayed^1^. After re-expressing exogenous GON-4 protein, fertility can be rescued in *gon-4* mutants. In *Drosophila*, the *Gon4l* homologous gene, *muscle wasted* (*mute*), has been proposed as one of the components of histone locus body. Loss of function in *mute* gene results in decreased muscle mass and defective terminal differentiation of muscle cells, therefore, suggesting a role for Mute in regulating muscle cell differentiation through controlling histone expression^2^.

For mammals, *Justy* mutant mice, which carry a point mutation within the *Gon4l* gene, exhibit defects in B cell development^3^. Detailed analysis reveals that a block in the G1/S phase transition results in severely defective differentiation of pre-pro B cells to pro-B cells in these mutant mice^4^. Moreover, a frameshift mutation in this gene causes proportionate dwarfism in Fleckvieh cattles^5^, indicating that the Gon4l protein not only affects cell proliferation/differentiation in specific types of tissues but also can impair the growth of an entire organism.

The zebrafish mutant, *ugly duckling* (*udu*^*tu24*^), was first isolated from the Tübingen large-scale screen and exhibits fewer blood cells, a shorter body axis, and defective tail formation^6^. Another study shows that *udu*^*sq3*^ mutants (originally named *udu*^*sq1*^) exhibit arrested primitive hematopoiesis^7^. The third *udu* mutant allele, *udu*^*vu66*^, shows an irregular notochord boundary and reduced mediolateral cell polarity at early stages^8^. Similar to human and mouse GON4L proteins, zebrafish Udu protein is also a nuclear factor. Its N-terminus contains three conserved regions (CR1, CR2, and CR3), and its C-terminus consists of two paired PAH (amphipathic α-helix like) repeats and one SANT domain (SWI3, ADA2, N-CoR, and TFIIIB like)^7^. The PAH domain, which has been identified in the *Saccharomyces cerevisiae SIN3* gene, has the ability to mediate protein–protein interaction^9,10^; and the SANT domain, which is similar to the Myb DNA-binding domain^11^, may be responsible for regulating chromatin remodeling and accessibility^12,13^. Therefore, the protein structure of human/mouse GON4L and zebrafish Udu indicates their roles in regulating gene expression through their incorporation with other proteins.

Despite the biological importance of Gon4l/Udu, only a few studies have addressed the biochemical interactions between Gon4l/Udu and other proteins, as well as how interactions among these proteins are related to its possible functions. GON4L in mouse B cells can associate with YY1, Sin3a and Hdac1 to form a complex. The interaction among these proteins correlates with the ability of GON4L to repress targeted DNA expression as determined by GAL4-UAS reporter assay^14^. GON4L in human and Mute in *Drosophila* are reported to be co-localized with NPAT (Nuclear Protein, Ataxia-Telangiectasia Locus) in the histone locus body to regulate the expression and processing of cell cycle-regulated histone transcripts^15,16^. Transcription levels of histones H3 and H4 are both up-regulated in *mute* mutant embryos, suggesting that Mute might be responsible for the repression of histone mRNA accumulation^2^. Additionally, a recent study using a large-scale protein–protein interaction screen in mouse T cells revealed a novel Gon4l-interacting protein, CRAMP1L, in association with the Hippo kinase, MST1^17^. However, whether the interaction among GON4L, CRAMP1L, and MST1 affects gene transcription in regulatory T cells was not further addressed. In contrast to being a transcriptional repressor, GON4L has been shown to interact with YY1 to positively regulate CD24 expression in several human cancer cell lines^18^. Noteworthy, it is very interesting that Gon4l/Udu may not only exert its function as a transcriptional regulator. Expression of the PAH/SANT domain in synchronized human cells shows that it is co-localized with the BrdU signal in early S phase and with HP1b, a marker for pericentromeric heterochromatin, in late S phase^19,20^. These findings suggest a possible function of Gon4l/Udu for participating in DNA replication and also in protecting the epigenetic integrity.

To further understand the possible functions of Gon4l/Udu, we performed yeast two-hybrid (Y2H) screens in this study to discover novel Gon4l/Udu-interacting proteins from prey libraries of both mouse cell lines and zebrafish larvae. We have isolated 56 Gon4l/Udu-interacting candidate proteins and used co-immunoprecipitation to verify 11 of them. We have identified four proteins as novel partners for Gon4l/Udu—Bap1, Dnmt1, Thoc1, and Cry3a—which are functionally related to histone modification, maintenance of DNA methylation after replication, R-looping prevention and RNA processing during transcription, and circadian-regulated DNA repair, respectively. RNA in situ hybridization studies show that *bap1*, *dnmt1*, *thoc1*, and *cry3a* co-localize with *gon4l*/*udu* during development mainly in the brain region and some other specific tissues. Furthermore, using bioinformatic analysis reveals that enriched phenotypes in mutant animals carrying mutations at genes encoding Gon4l/Udu-interacting proteins are similar to those observed in reported *Gon4l*/*udu* mutants. These data not only indicate functional cooperation among Gon4l/Udu and its interacting proteins but also provide important links between the molecular functions of Gon4l/Udu and various kinds of reported biological effects.

## Results

### 56 zebrafish Udu and mouse GON4L interacting candidates are identified by yeast 2-hybrid analyses

**Figure 1:**
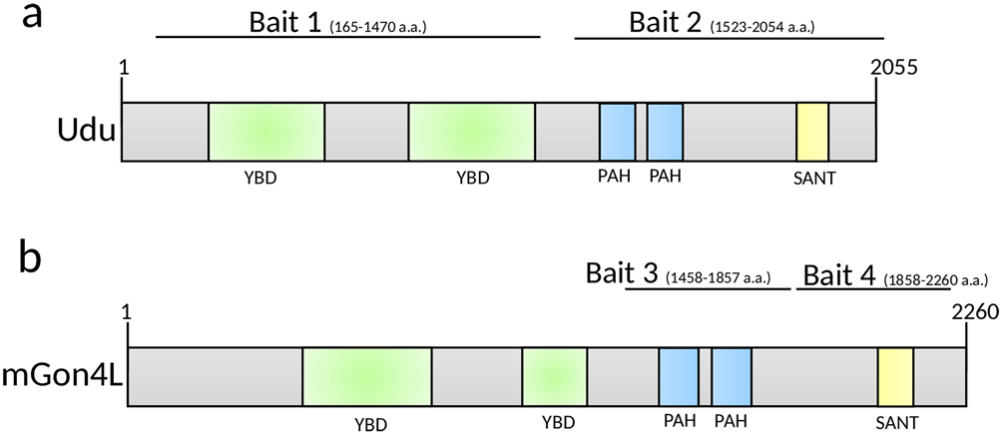
Schematic structures of the bait proteins. Domain structures of (**a**) zebrafish Udu and (**b**) mouse GON4L. Black lines indicate locations of bait used in this study.

In order to understand the functions of Gon4l/Udu, we searched for novel proteins interacting with Udu in zebrafish and GON4L in mouse using the yeast two-hybrid system. Two fragments of zebrafish Udu—including the N-terminal CR1-CR3 (bait 1: 165–1,470 a.a.), which contains two putative YY1-binding domains, and the C-terminal PAH and SANT domains (bait 2: 1,523–2,054 a.a.)—were used as bait (Fig. 1a) to screen the zebrafish embryonic cDNA library. We also used two fragments for mouse GON4L, including the PAH domains (bait 3: 1,458–1,857 a.a.) and the SANT domain (bait 4: 1,858–2,260 a.a.), to screen different murine cDNA libraries originated from embryos, B lymphocytes (B cells) and cardiac myocytes (Fig. 1b). In the zebrafish screen, 15 and 11 proteins were identified by bait 1 and bait 2, respectively (Table 1). Mcm3 and Mcm4, identified by the latter, have been previously reported^19^. In the mouse screen, 29 proteins were identified as interacting with bait 3 (Table 2); among them, CRAMP1L has been shown to interact with GON4L in both human and mouse models^21^. The only protein identified by bait 4 was Npat (Table 2), which was reported as having the ability to interact with Gon4l in human and *Drosophila*^16^.

**Table 1:** Zebrafish Udu-binding proteins isolated by yeast two-hybrid analysis.

Category	Bait	PBS*	Prey binding region	Symbol	Name (accession number)	GO terms
Lipid metabolism-related	1	D	unknown-3786	Apobb.1	Apolipoprotein Bb, tandem duplicate 1 (apobb.1), mRNA (NM_001030062.1 )	Lipid transporter activity
	1	D	− 1 ~ 399	Ephx2	Epoxide hydrolase 2, cytoplasmic, transcript variant X1, mRNA (XM_021467153.1)	Epoxide hydrolase activity
Transcriptional regulation	1	D	432 ~ 1,248	Pax7b	Paired box 7b (NM_001146149.1)	Sequence-specific DNA binding
	2	D	92 ~ 652	Hoxc4a	Homeobox C4a, mRNA (NM_131122.2)	Sequence-specific DNA binding
	1	D	96 ~ 600	Otx1	Orthodenticle homeobox 1, mRNA (NM_131250.2)	Sequence-specific DNA binding
	1	D	− 1 ~ 954	Cry3a	Cryptochrome circadian regulator 3a, transcript variant 1, mRNA (NM_001313822.1)	Negative regulation of transcription; sequence-specific DNA binding
	2	D	− 7 ~ 754	Pelp1	Proline, glutamate and leucine rich protein 1, mRNA (XM_001338982.7)	Transcription factor binding; chromatin binding
RNA-binding/processing	1	D	2094 ~ 2,781	Heatr6	HEAT repeat containing 6 (NM_001114313.1)	RNA binding
	2	D	− 1 ~ 614	Fbrsl1	Fibrosin-like 1 protein variant 1a mRNA, complete cds, alternatively spliced (KY492379.1)	RNA binding
	2	D	− 1 ~ 1,026	Rbmx2	RNA binding motif protein X-linked 2, mRNA (NM_001025166.1)	RNA binding
Apoptosis/Cell proliferation/DNA replication	2	D	1,155 ~ 1893	Mcm3	Minichromosome maintenance complex component 3, mRNA (NM_212567.2)	DNA replication origin binding; helicase activity
	2	D	1971 ~ 2,229	Mcm4	minichromosome maintenance complex component 4, mRNA (NM_198913.2)	DNA replication origin binding; helicase activity
	1	D	− 1 ~ 1,355	Bnipl	BCL2/adenovirus E1B 19kD interacting protein, like, transcript variant X1, mRNA (XM_009292526.3)	Exopolyphosphatase activity
	2	D	270 ~ 1744	Gulp1b	GULP, engulfment adaptor PTB domain containing 1b, transcript variant X5, mRNA (XM_001345116.6)	Apoptotic process
	2	D	135 ~ 706	Gpatch11	G patch domain containing 11, mRNA; zgc92714 (NM_001002393.1)	Nucleic acid binding
Signaling pathways	1	D	1,356 ~ unknown	Dlc	deltaC, mRNA (NM_130944.1)	PDZ domain binding; Notch binding
	1	D	Unknown ~ 1,216	Napba	N-ethylmaleimide-sensitive factor attachment protein, beta, mRNA (NM_001020655.2)	Soluble NSF attachment protein activity; syntaxin binding
	2	D	2,640 ~ 3,309	Dock6	Danio rerio dedicator of cytokinesis 6, transcript variant X1, misc_RNA (XR_660514.3)	Guanyl-nucleotide exchange factor activity
Protein translocation/ processing	1	D	Unknown ~ 915	Ssr1	Signal sequence receptor, alpha, mRNA (NM_201327.1)	Cellular response to estrogen
	1	D	− 1 ~ 559	Naa15b	N(alpha)-acetyltransferase 15, NatA auxiliary subunit b, mRNA (NM_203321.2)	Peptide alpha-N-acetyltransferase activity
Cytoskeleton	1	D	− 1 ~ 628	Agrn	Agrin, mRNA (NM_001177452.1)	Serine-type endopeptidase inhibitor activity
	1	D	− 1 ~ 608	Pleca	plectin a, mRNA (NM_001100032.1)	Ankyrin binding; actin binding
	1	D	147 ~ 841	Actc1	cardiac muscle alpha actin 1 (NM_001002066.1)	Nucleotide binding; ATP binding
	2	D	− 1 ~ unknown	Neb	Nebulin (neb), transcript variant X41, mRNA (XM_021474182.1)	Actin filament binding
	2	D	− 1 ~ unknown	Cnn3b	Calponin 3, acidic b, mRNA (NM_001024073.1)	Actin binding; calmodulin binding
Others	1	D	225 ~ 661	Slc25a3b	Solute carrier family 25 (mitochondrial carrier; phosphate carrier), member 3b, mRNA (NM_213722.1)	Inorganic phosphate transmembrane transporter activity

*PBS is a score that is automatically computed through algorithms; D means “moderate confidence in the interaction”.

**Table 2:** Mouse GON4L-binding proteins isolated by yeast two-hybrid analysis.

Category	Bait	Prey binding region	Prey library	Symbol	Name (accession number)	GO terms
Lipid metabolism-related	3	16 ~ 240	Mouse embryo	GC	Group specific component (NM_008096)	Vitamin D-binding; vitamin transmembrane transporter activity
	3	− 3 ~ 374 − 13 ~ 97 3 ~ 374	Mouse B cell/Cardiac cell	LIAS	Lipoic acid synthetase, mitochondrial protein (NM_024471)	Lipoate synthase activity; transferase activity
Transcriptional regulation	3	5 ~ 176	Mouse embryo	FOXA2	Forkhead box a2 (NM_010446)	DNA-binding transcription factor activity
	3	189 ~ 547	Mouse B cell	HIVEP1	Human Immunodeficiency Virus Type I Enhancer Binding Protein 1 (NM_007772)	DNA-binding transcription factor activity
	3	259 ~ 580	Mouse B cell	ARNTL2	Aryl hydrocarbon receptor nuclear translocator-like 2, also known as Bmal2 (NM_172309)	DNA-binding transcription factor activity
	3	821 ~ 1,006	Mouse B cell	BRD4	Bromodomain containing 4 (NM_020508)	Lysine-acetylated histone binding; chromatin binding
	4	1,233 ~ 1,421 1,355 ~ 1,421	Mouse Cardiac cell	NPAT	Nuclear Protein, Ataxia-Telangiectasia Locus (NM_001081152)	Regulation of transcription involved in G1/S transition of mitotic cell cycle; transcription coactivator activity of histones
	3	654 ~ 897	Mouse B cell	CRAMP1L	Cramped-like, cramp1L (NM_020608)	DNA binding; nucleus; chromatin binding
	3	591 ~ 726	Mouse Cardiac cell	SKI	ski sarcoma viral oncogene homolog (NM_011385)	SMAD binding; transcription corepressor activity
	3	118 ~ 296	Mouse B cell	TLE3	Transducin-Like Enhancer Of Split 3, Homolog Of Drosophila E (NM_009389)	Transcription corepressor activity
RNA-binding/processing	3	338 ~ 532	Mouse embryo	THOC1	Tho complex 1, also known as Tho1, HPR1 (NM_153552)	DNA binding; RNA binding; protein binding; nucleus; cytoplasm; mRNA export from nucleus; RNA splicing; replication fork processing
	3	− 6 ~ 126 − 3 ~ 126 8 ~ 97 24 ~ 118	Mouse Cardiac cell/Mouse embryo	RPS25	Ribosomal Protein S25 (NM_024266)	Structural constituent of ribosome
	3	401 ~ 655 431 ~ 664 435 ~ 711	Mouse B cell	DDX10	DEAD-Box Helicase 10 (XM_284494)	RNA binding; RNA helicase activity; ATP binding
	3	74 ~ 458 200 ~ 477 26 ~ 479 373 ~ 511	Mouse B cell/ Mouse embryo	CTNNBL1	Beta-catenin-like protein 1 (NM_025680)	mRNA splicing via spliceosome; protein binding; positive regulation of apoptotic process
Apoptosis/Cell proliferation/DNA replication	3	625 ~ 729 545 ~ 646	Mouse B cell/ Mouse embryo	BAP1	BRCA1-associated protein 1 (NM_027088)	Chromatin binding; thiol-dependent ubiquitinyl hydrolase activity
	3	− 21 ~ 202	Mouse embryo	CACYBP	Calcyclin binding protein (NM_009786)	Tubulin binding; ubiquitin protein ligase binding; S100 protein binding
	3	423 ~ 715	Mouse Cardiac cell	CIT	Citron Rho-interacting Kinase (NM_007708)	Protein serine/threonine kinase activity; mitotic cytokinesis
	3	554 ~ 831 574 ~ 704	Mouse B cell/Cardiac cell	DNMT1	DNA methyltransferase 1 (AF162282)	Chromatin binding; DNA (cytosine-5-)-methyltransferase activity
	3	254 ~ 656 547 ~ 643	Mouse B cell/Cardiac cell	KIF20A	rabkinesin-6 (Y09632)	ATPase activity; microtubule motor activity; nucleotide binding
Signaling pathways	3	48 ~ 435 313 ~ 439 339 ~ 446 262 ~ 461 340 ~ 558 250 ~ 465	Mouse Cardiac cell/ Mouse embryo	GPC1	Glypican-1 (NM_016696)	Fibroblast growth factor binding
	3	65 ~ 582	Mouse embryo	ITSN1	Intersectin 1 (NM_010587)	Guanyl-nucleotide exchange factor activity; Rho guanyl-nucleotide exchange factor activity; calcium ion binding
	3	410 ~ 526	Mouse embryo	PPP3CB	Protein phosphatase 3, catalytic subunit, beta isoform (NM_008914)	Calmodulin-dependent protein phosphatase activity
	3	249 ~ 554	Mouse B cell	FCHSD2	FCHSD2/Carom/FCH and double SH3 domains 2/F-BAR And Double SH3 Domains Protein 2 (NM_001146010)	Phosphatidylinositol-3,4,5-trisphosphate binding
	3	367 ~ 523	Mouse Cardiac cell	STAM1	Signal-Transducing Adaptor Molecule 1 (NM_011484)	Ubiquitin-like protein ligase binding
	3	242 ~ 419 251 ~ 419 269 ~ 419	Mouse B cell/Mouse embryo	ALS2CR2	STE20-related kinase adapter protein beta; STRADB (NM_172656)	Protein serine/threonine kinase activity; ATP binding
	3	4 ~ 209	Mouse Cardiac cell	TNIP3	TNFAIP3 Interacting Protein 1, A20-binding inhibitor of NF-κB activation (ABIN-1) (NM_001001495)	Protein deubiquitination; identical protein binding; MAPK binding
	3	259 ~ 554	Mouse B cell	TNIP1	TNFAIP3 Interacting Protein 3), A20-binding inhibitor of NF-κB activation (ABIN-3) (NM_021327)	Protein deubiquitination; identical protein binding; MAPK binding
Protein processing/translocation	3	− 37 ~ 50	Mouse embryo	AHSA1	AHA1, Activator of Heat Shock 90 kDa Protein ATPase Homolog 1 (NM_146036)	ATPase activator activity; Hsp90 protein binding; extracellular exosome
	3	3 ~ 279 52 ~ 279 − 5 ~ 279 14 ~ 279	Mouse B cell/Cardiac cell/Mouse embryo	C1QBP	Complement component 1 Q subcomponent-binding protein, mitochondrial (NM_007573)	Mitochondrial ribosome binding; mRNA binding; translation activator activity
Others	3	269 ~ 376 302 ~ 383	Mouse embryo	KLHDC3	Kelch domain containing 3,transcript 1 (NM_027910)	Chromatin binding; meiotic recombination

### Bap1, Dnmt1, Thoc1, Cry3a, Bnipl, Brd4, Foxa2 and Kif20a are co-localized with Udu in the nucleus

According to the categories of gene ontology (GO, ref.^22,23^) and the known functions of Udu, eleven genes from both mouse and zebrafish Y2H data (Table 1 and 2) were selected for co-immunoprecipitation (co-IP) analysis to confirm whether these candidate proteins are indeed Gon4l/Udu-interacting proteins. Among those selected, eight were related to replication fork processing, histone/DNA modifications, or transcription processing. The other three were chosen because of their metabolic functions that have never been connected to Gon4l or Udu.

**Figure 2:**
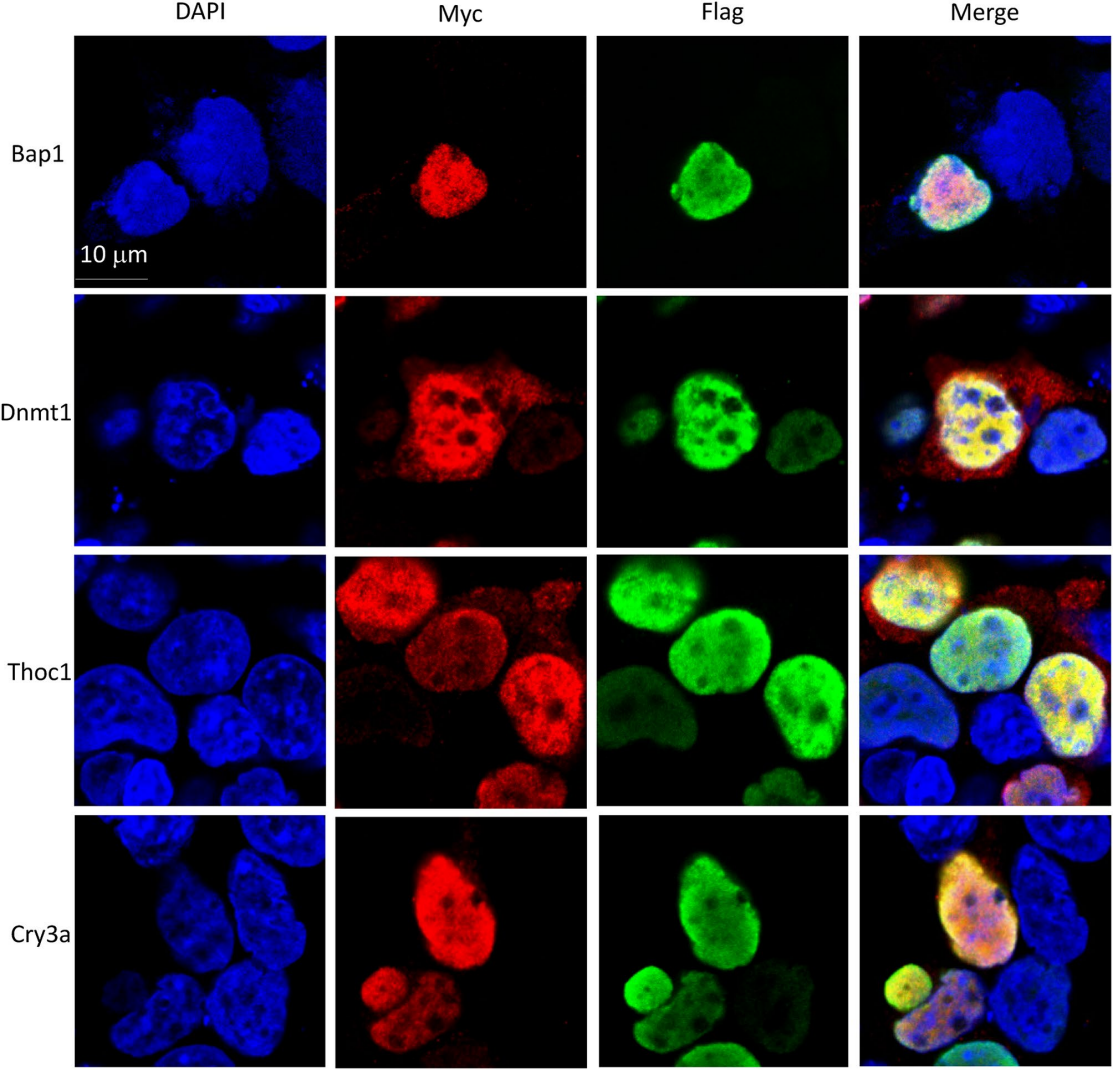
Confocal images for the cellular co-localization analysis of Udu and its interacting proteins. For co-localization analysis, Flag-tagged Udu-P/S construct was co-transfected individually with each Myc-tagged construct, including Bap1, Dnmt1, Thoc1, and Cry3a. Anti-FLAG M2 monoclonal antibody and rabbit anti-MYC antibody were used. Alexa Fluor 488-conjugated anti-mouse secondary antibody and Alexa Fluor 564-conjugated anti-rabbit secondary antibody were then applied. Green fluorescence indicates Udu-P/S, while red fluorescence indicates the expression of Myc-tagged proteins. Blue color is DAPI used for nuclear counterstain. Merged images show the nuclear co-localization of Udu and the interacting proteins.

Because the important domains of mouse GON4L and zebrafish Udu are highly conserved^3,7^, we examined the interactions between zebrafish Udu and the zebrafish counterparts of selected proteins identified by different Y2Hs. We first cloned those candidate genes from zebrafish adult liver or embryonic cDNAs and fused them with Myc tag. Full-length zebrafish Udu (hereafter Udu-full) and Udu with only the PAH/SANT domain (hereafter Udu-P/S) were fused with Flag tag. Since Udu is known as a nuclear factor^7,19^, nuclear localization of Udu-full or Udu-P/S was first confirmed using immunofluorescence (Supplementary Fig. S1). Next, we co-transfected Flag-tagged Udu-full or Udu-P/S expressing constructs with Myc-tagged candidate protein constructs and performed double immunofluorescence staining and confocal microscopy to investigate their cellular localizations (Fig. 2 and Supplementary Fig. S2,S3). As compared to Udu-P/S, the transfection efficiency for Udu-full was relatively low. Therefore, we detected only a few cells co-expressing Udu-full and Myc-tagged candidate interacting proteins (Supplementary Fig. S2). We found that BRCA1-associated protein 1 (Bap1), DNA methyltransferase 1 (Dnmt1), Tho complex 1 (Thoc1), and Cryptochrome circadian regulator 3a (Cry3a) (Fig. 2 and Supplementary Fig. S2), as well as Bromodomain containing 4 (Brd4), Forkhead box a2 (Foxa2), and Rabkinesin-6 (Kif20a) were all co-localized with Udu-P/S in the nucleus (Supplementary Fig. S3). In addition to the major localization of Dnmt1 and Thoc1 in the nucleus, we also detected their lower expressions in the cytoplasm (Fig. 2), which are similar to previous studies^24–26^. Two alternative splice forms of human BCL2/adenovirus E1B interacting protein like (BNIPL), BNIP-Sα and BNIP-Sβ, have been reported to be expressed in the cytoplasm and nucleus, respectively^27^. Interestingly, we found some cells expressed both cytoplasmic and nuclear Bnipl (Supplementary Fig. S3), and therefore is co-localized with Udu in the nucleus. For those metabolism-related proteins, including Epoxide hydrolase 2 (Ephx2), Group specific component (Gc), and Lipoic acid synthetase (Lias), showed only cytoplasmic localization and are therefore unlikely to interact with Udu (Supplementary Fig. S3). Taken together, these results identified eight candidate proteins that may interact and cooperate with Udu in the nucleus.

### Four newly identified interacting proteins (Bap1, Dnmt1, Thoc1, and Cry3a) indicate novel functions of Gon4l

**Figure 3:**
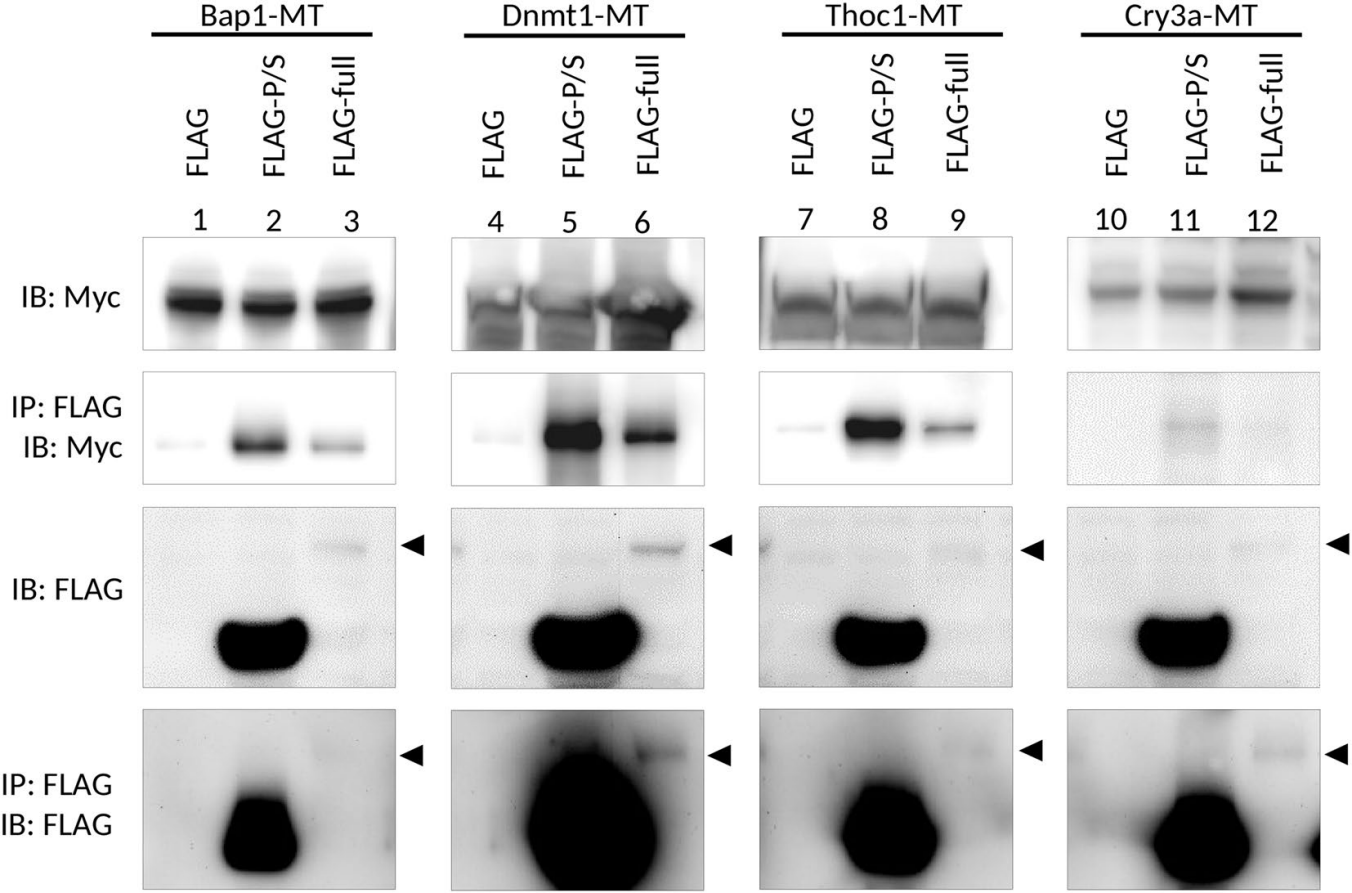
Co-immunoprecipitation analysis for the interaction of Udu with Bap1, Dnmt1, Thoc1, and Cry3a. Flag-tagged control or Flag-tagged Udu-expressing constructs were co-transfected with different Myc-tagged candidate Udu-interacting proteins, including Bap1, Dnmt1, Thoc1, and Cry3a. The cell lysate was incubated with anti-FLAG M2 magnetic beads, then subjected to immunoblot with anti-MYC or anti-FLAG antibody. Arrowheads indicate the expected size of full-length Udu. Lanes 1 to 9 were cropped from the same blots, while lanes 10–12 were from the other ones. To observe the expression of full-length Udu, photographs using a longer exposure time of anti-FLAG immunoblots were taken. The original un-cropped immunoblots were shown in Supplementary Fig. S7a and photographs with shorter exposure time for anti-FLAG immunoblots were also included in Supplementary Fig. S7b as supporting information.

To analyze the direct interactions of these candidate proteins with Udu, the whole cell lysate was immunoprecipitated by anti-FLAG antibody and then subjected to Western blot analysis by anti-FLAG or anti-MYC antibody to detect whether the candidate proteins were co-immunoprecipitated with Udu-full or Udu-P/S. Only Bap1, Dnmt1, and Thoc1 proteins showed strong signals with Udu-P/S (Fig. 3, lane 2, 5, and 8). The Udu-P/S-binding affinity is weaker for Cry3a (Fig. 3, lane 11). Notably, although signals for both the expression level and the amount of immuoprecipitated Udu-full were very weak, milder interaction signals between Udu-full and Bap1, Dnmt1 or Thoc1 could still be detected (Fig. 3, lane 3, 6, and 9). Interaction signals for Udu-full and Cry3a were barely detectable (Fig. 3, lane 12). No co-IP signal for the other candidate proteins, including Bnipl, Brd4, Foxa2, Kif20a, Ephx2, Gc, and Lias, was detected (data not shown).

In addition to identifying proteins by co-IP experiments in this study, we also searched for more Gon4l-interacting proteins to get a clearer picture of multi-functional Gon4l protein. The Biological General Repository for Interaction Datasets (BioGRID) is a database that collects and archives protein, genetic, and physical interactions for model organisms, such as mice and humans (https://thebiogrid.org). Therefore, we used BioGRID to find Gon4l-interacting proteins that were identified from large-scale screenings but had never been specifically addressed in the literature. The dataset from mouse studies showed only 3 known Gon4l-interacting proteins, including YY1, Sin3A and Hdac1, all of which can co-regulate transcription of downstream genes (Supplementary Fig. S4a)^14^. We noticed that among ten physically interacting proteins in the datasets from humans, five belong to the histone protein family and two are related to transcriptional regulation (Supplementary Fig. S4b and Supplementary Table S1). This finding, together with our data, reinforces the role of Gon4l in regulating chromatin remodeling as well as gene expression.

### Prediction for enriched phenotypes of Gon4l/Udu-interacting proteins leads to similar phenotypes observed in *Gon4l/udu* mutant animals

We then asked whether the phenotypic changes observed in *Gon4l/udu* mutant animals are correlated to the biological functions of its interacting proteins. Therefore, we applied model organism Phenotype Enrichment Analysis (modPhEA, Ref.^28^) to analyze phenotypes that are enriched by 20 Gon4l-interacting proteins (including those reported in the literature, ten other physically interacting proteins found in BioGRID datasets, and novel proteins identified in this study). The proteins used for modPhEA are listed in Supplementary Table S2. Among those genes input, five genes were ignored, because of the lack of annotated phenotypes in the database. According to the mouse phenome database from modPhEA (Gene Expression Database (GXD), Mouse Genome Informatics Web Site, MGI6.14, https://www.informatics.jax.org), the most enriched phenotypes are related to increased embryonic tissue cell apoptosis (p = 0.028), embryonic lethality (p = 0.014), increased T cell derived lymphoma incidence (p = 0.006), decreased cell proliferation (p = 0.012), chromosome instability (p = 0.018), and abnormal dopamine level (p = 0.014) (Table 3 and Supplementary Table S3). After focusing on Bap1, Dnmt1, Thoc1, and Cry3a identified in this study, we noticed that Bap1, Dnmt1, and Thoc1 were most related to increased embryonic tissue cell apoptosis (p = 0.001) (Supplementary Table S4). However, Cry3a did not account for the enrichment of apoptotic phenotype, indicating the specific importance of Bap1, Dnmt1, and Thoc1 proteins to cell survival during development (Supplementary Table S4). Additionally, we also analyzed the phenotype enrichment using the *Caenorhabditis elegans* database from modPhEA (WBPhenotype, data version: WormBase web site, https://www.wormbase.org, release WS273, November 2019). Similar to results obtained from mouse phenome, early embryonic lethal was enriched (p = 0.001) in the WBPhenotype database. Other phenotypes—such as nuclear morphology variation early emb (p = 1.2 × 10^–5^), sister chromatid segregation defective early emb (p = 6.3 × 10^–5^), embryonic cell morphology variant (p = 3.6 × 10^–4^), body elongation variant (p = 0.002), programmed cell death variant (p = 0.004), thin (p = 0.004), and reproductive system morphology variant (p = 0.007)—were also enriched by Gon4l-interacting proteins (Supplementary Table S5). These results were consistent with the reported phenotypes found in *Gon4l*/*udu* mutant organisms.

**Table 3:** List of the most enriched phenotypes and their corresponding Udu/Gon4l-interacting genes after model organism Phenotype Enrichment Analysis of mouse phenome database.

Phenotype name	BH FDR corrected P-value	Genes with the term
**Embryo phenotype**	0.023	mcm4, npat, thoc1, bap1, dnmt1, yy1, hdac1
Increased embryonic tissue cell apoptosis	0.028	thoc1, dnmt1
**Mortality/aging**		
Prenatal lethality	0.012	mcm3, mcm4, thoc1, bap1, dnmt1, yy1, sin3a, hdac1
Embryonic lethality, complete penetrance	0.007	mcm4, dnmt1, yy1, hdac1
Embryonic lethality between implantation andsomite formation, complete penetrance	0.036	mcm4, sin3a
**Neoplasm**		
Increased hemolymphoid system tumor incidence	0.007	mcm4, esr2, h2afx
Increased T cell derived lymphoma incidence	0.006	mcm4, h2afx
**Cellular phenotype**		
Abnormal cell proliferation	0.022	mcm3, mcm4, dnmt1, esr2, h2afx, hdac1
Decreased cell proliferation	0.012	mcm3, mcm4, dnmt1, h2afx, hdac1
Chromosomal instability	0.018	mcm4, h2afx
**Nervous system phenotype**		
Abnormal dopamine level	0.014	esr2, pnkd

### Analysis for the mRNA expression of Gon4l/Udu-interacting proteins, Bap1, Dnmt1, Thoc1, and Cry3a

**Figure 4:**
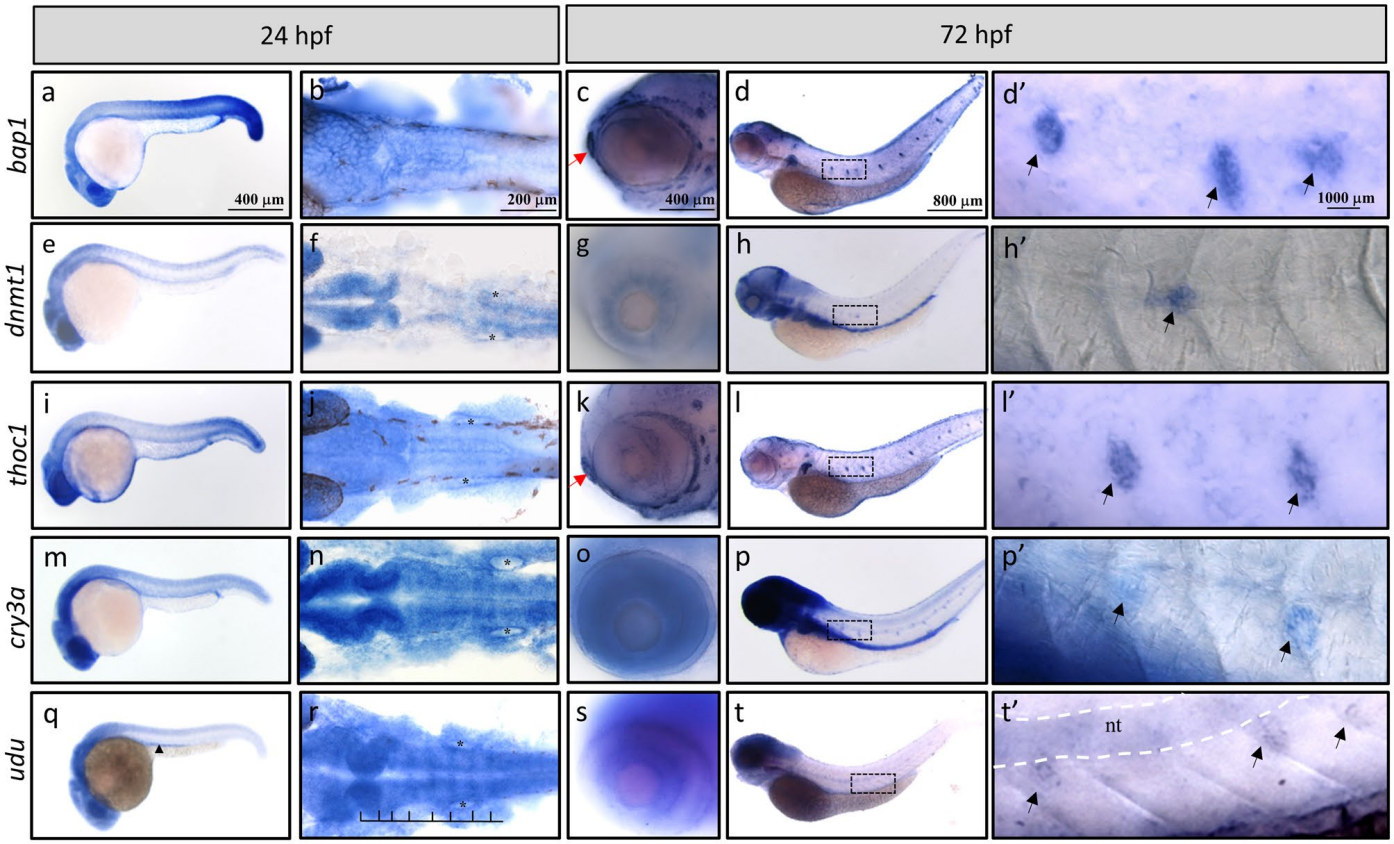
Whole mount in situ hybridization analysis for temporal and spatial expression of the *bap1*, *dnmt1*, *thoc1*, *cry3a*, and *udu* genes during zebrafish early development. WISH of (**a**–**d’**) *bap1*, (**e**–**h’**) *dnmt1*, (**i**–**l’**) *thoc1*, (**m**–**p’**) *cry3a*, and (**q**–**t’**) *udu* expression at 24 hpf (**a**,**b**,**e**,**f**,**i**,**j**,**m**,**n**,**q**,**r**) and 72 hpf (**c**,**d’**,**g**,**h’**,**k**,**l’**,**o**,**p’**,**s**,**t’**) embryos. Black arrowhead indicates the pronephric duct (**q**). Rhombomere boundaries are indicated by black lines (**r**). Black asterisks indicate otic vesicles (**f**,**j**,**n**,**r**). Red arrows indicate olfactory sensory epithelium (**c**,**k**). Dashed rectangles in (**d**,**h**,**l**,**p**,**t**) are shown as enlarged images in (**d’**,**h’**,**l’**,**p’**,**t’)**, respectively. Black arrows indicate neuromasts of the posterior lateral line (**d**’,**h’**,**l’**,**p’**,**t’**) and white dash lines indicate notochord (**t’**). Flat mount of WISH samples: (**b**,**f**,**j**,**n**,**r**). All embryos are oriented with anterior to the left.

The above analyses indicated that the major biological functions of Gon4l/Udu may be mediated through these interacting proteins. For example, Bap1, Dnmt1, and Thoc1 are highly associated with increased embryonic tissue cell apoptosis and prenatal lethality (Table 3 and Supplementary Table S4). These enriched phenotypes are similar to the extensive amount of apoptotic cells and subsequent embryonic lethality observed in *udu*^*tu24*^ mutants^6,19^, indicating a consequence from the loss of Udu interactions with Bap1, Dnmt1, and/or Thoc1. To further verify this hypothesis, we examined the mRNA expression patterns of *bap1*, *dnmt1*, and *thoc1* during development. Due to the lack of reported phenotypes about *Cry3a* mutants in the modPhEA databases, we also checked the expression patterns of *cry3a* and compared those of four with that of *udu* at the same stages (Fig. 4 and Supplementary Fig. S5). We performed in situ hybridization for *bap1* (Fig. 4a–d’ and Supplementary Fig. S5a–d), *dnmt1* (Fig. 4e–h’ and Supplementary Fig. S5e–h), *thoc1* (Fig. 4i–l’ and Supplementary Fig. S5i–l), and *cry3a* (Fig. 4m–p’ and Supplementary Fig. S5m–p). Surprisingly, expression patterns of these four genes were very similar to each other. All four genes were ubiquitously expressed at 6 h post fertilization (hpf) (Supplementary Fig. S5a,e,i,m), and became specifically expressed in the CNS, eyes, and tail region at 24 hpf (Fig. 4a,b,e,f,i,j,m,n). *dnmt1, thoc1,* and *cry3a*, different from *bap1*, are additionally expressed in the epithelium of otic vesicles (Fig. 4f,j,n). At 48 hpf, the signals in the trunk were getting weaker, while their expressions in the brain region remained strong (Supplementary Fig. S5c,g,k,o). Furthermore, *bap1* and *thoc1* started to be detected in the cranial neuromasts and olfactory sensory epithelium (Fig. 4c,k). All four genes were detected in eyes, pectoral fin buds, and neuromasts of the posterior lateral line at 48 hpf and 72 hpf (Fig. 4c,d’,g,h’,k,l’,o,p’, and Supplementary Fig. S5c,d,g,h,kl,o,p). Besides, strong expression signals of *dnmt1* and *cry3a* were detected in the developing gut at 48 and 72 hpf (Fig. 4h,p, Supplementary Fig. S5g,h,o,p). Our observations for the expression patterns of *dnmt1* mRNA were very similar to the previously reported expression patterns that restricted to the brain region, retina, branchial arches, pectoral fin buds, lateral line system and digestive system at later time points^29,30^. *cry3a* mRNA expression has been reported to be expressed in the brain, ganglion cell layer of retina, and the liver of zebrafish larvae^31^, as well as in the brain, the retina, the liver, and muscles in the adult zebrafish^31,32^. Our study presents here for the first time that in addition to those reported tissues, *cry3a* is also expressed in pectoral fin buds, the lateral line system and the digestive system (including liver and intestine) during development.

We found that, similar to previous reported observations, *udu* mRNA was expressed in a ubiquitous manner at early stages (Supplementary Fig. S5q). At 24 hpf, *udu* expression was detected in the CNS, eyes, and pronephric duct (Fig. 4q,r). We also noticed a boundary-specific expression of *udu* in the rhombomeres (Fig. 4r) at 24 hpf. Similar to *dnmt1*, *thoc1*, and *cry3a*, *udu* expression was detected in the otic vesicles (Fig. 4f,j,n,r). At 48 and 72 hpf, *udu* started to be detected in retina and weakly expressed in the notochord (Fig. 4s,t and Supplementary Fig.q S5s,t). Moreover, *udu* was also detected in neuromasts of the posterior lateral line (Fig. 4t–t’). Therefore, these data further indicate that during development *udu* is largely co-expressed with *bap1*, *dnmt1*, *thoc1*, and *cry3a*, all of which cooperate with each other to maintain cell survival in a variety of tissues, such as the embryo proper of early stages, CNS, eyes, and neuromasts.

## Discussion

The Udu protein in zebrafish, as well as its homologs among species, is a nuclear factor that possesses pleiotropic functions and influences the differentiation and proliferation of various cell types. Gon4l can collaborate with YY1, SIN3A and HDAC1 to repress gene expression and has been suggested to act as a co-regulator for transcription or as a platform for transcriptional complex formation^14^. In addition to direct regulating gene expression, Gon4l may also be important for pathways related to cell division and maintenance of genomic stability^19^, but how Gon4l/Udu exerts its function is still unclear. In this study, we used Y2H to identify novel Gon4l/Udu-interacting proteins, which is further confirmed by co-IP analysis to clarify the possible unknown functions of Gon4l/Udu. So far, we have identified four novel Gon4l/Udu-interacting proteins: Bap1, Dnmt1, Thoc1, and Cry3a. Interestingly, we perceived that zebrafish and mouse Y2Hs isolated non-overlapping Gon4l/Udu-interacting candidates. The lack of overlap may be due to the following reasons. First, the screening scale of the zebrafish Hybrigenics Y2H is about 5 to 10 folds higher than that of mouse Myriad Genetics. Second, the zebrafish prey library is made only from embryos; in contrast, three different mouse prey libraries are made from embryos, B cells, and cardiac cells, respectively. Third, and most importantly, the Hybrigenics system uses fragmented cDNAs for constructing prey library, while that of Myriad Genetics basically constructs the prey libraries with full-length cDNAs. This distinction gives rise to the differences in expressivity, toxicity, and interactivity of prey proteins.

It has been demonstrated that DNA double-strand break (DSB)-induced DNA damage response (DDR) is activated in *udu*^*tu24*^ mutants^19^. However, the molecular mechanism of how Gon4l/Udu induces DDR remains unknown. The nuclear localized deubiquitylating enzyme BAP1^33,34^, which is responsible for histone modification in transcription regulation^35,36^, can be phosphorylated by Ataxia telangiectasia mutated (ATM) and participates in DNA double-strand break (DSB) repair through regulating histone 2A and H2AX ubiquitylation^37,38^. Moreover, according to the online database BioGRID, Gon4l/Udu physically interacts with H2AX and other histone subtypes (Supplementary Table S1). Based on these data and the newly identified interaction between Bap1 and Gon4l/Udu, Bap1 may provide a key connection between Gon4l/Udu and DDR.

The most abundant DNA methyltransferase, DNMT1, is considered a major enzyme for maintaining global DNA methylation in concert with other histone modifiers and regulates gene transcription and genomic integrity^39^. It has been shown that lack of *Dnmt1* expression leads to impaired self-renewal of hematopoietic stem cells (HSCs) in both adult mice and zebrafish models^40,41^. Down regulation of *cebpa* is crucial for the differentiation of pre-pro B cells to early pro-B cells^42–44^. In contrast to wild type, the mRNA level of *cebpa* is significantly up-regulated in the B cell progenitors of *Justy* mutant mice^3^. Further analysis using double morpholino injection revealed that Dnmt1 maintains HSCs and progenitor cells in zebrafish through regulating *cebpa* expression^40^. Similar defective hematopoietic phenotypes were also observed in *Gon4l*/*udu* mutants. The proliferation and differentiation of pre-pro B cells to early pro-B cells are impaired in *Justy* mutant mice^3,4^. In zebrafish, *udu*^*tu24*^ mutants exhibited fewer blood cells and defective proliferation and differentiation of erythrocytes were observed in *udu*^*sq3*^ mutants^6,7^. Although hematopoietic defects were not specifically described in the study using *udu*^*vu66*^ mutant embryos, its supplementary data using DamID-seq at the gastrula stage shows that the promoter region of *cebpa* was Udu-enriched, indicating that *cebpa* may be directly regulated by Udu^8^. Consistently, up-regulation of *cebpa* was observed in *udu*^*tu24*^ mutants at 22 hpf (Supplementarey Fig. S6) as compared to wild type. According to these findings, we propose that the interaction between Gon4l/Udu with Dnmt1 may be responsible for regulating *cebpa* expression and, therefore, play an important role during hematopoiesis.

Thoc1 protein is an essential component of Tho/Trex complex to restrain harmful R-loops during transcription^45,46^. A study of Thoc1-interacting proteins reveals that Thoc1 restrains R-loops not only through direct RNA-binding but also through cooperation with Sin3a to promote transient histone deacetylation after transcription^47^, which is consistent with the fact that Gon4l associates with Sin3a, HDAC1, and YY1 as a part of a complex to suppress gene expression at the transcriptional level^14^. Additionally, Gon4l/Udu also interacts with Mcm3 and Mcm4 during DNA replication^19^. Therefore, it may be important to elucidate whether Gon4l/Udu integrates with Mcm3/Mcm4, Sin3a and Thoc1 together during transcription and DNA replication to prevent R-loop formation as well as subsequent head-on transcription-replication collisions, genomic instability, and cell death.

According to the RNA in situ hybridization data from this study and previous reports, we have found that the expression pattern of *udu* is mainly similar to those of *dnmt1, bap1*, and *thoc1* genes, especially in the pectoral fin buds, eyes, CNS, and neuromasts of the posterior lateral line (Fig. 4). In addition to similar expression patterns of these genes, phenotypes including extensive apoptotic cells in head, neural tube, and tail, as well as decreased size of eyes and head have also been described in *udu*^6,19^, *dnmt1*^30,48^, *bap1*^49,50^, and *thoc1*^51^ knockdown or mutant animals. In *dnmt1* mutant zebrafish, defective lens with dysplasia have been observed^30^. Additionally, inherited mutations in human *DNMT1* have been associated with neurodegenerative syndromes that are characterized by degeneration of the cerebellum and of the acoustic and optic nerves^48^. In *Xenopus*, expression of *bap1* is restricted in the neural crest cells in early stages, and loss of *bap1* results in abnormal gastrulation and malformation of ocular structure^49^. Zebrafish embryos injected with *bap1* morpholino exhibited necrotic CNS^50^. Thoc1 homozygous null mouse is embryonic lethal due to higher apoptosis^51^. Moreover, results obtained from modPhEA analyses revealed the role of *dnmt1*, *bap1*, and *thoc1* in maintenance cell survival of embryos. According to the expression pattern, phenotypic analyses, and evidence for protein interactions, we suggest that *udu*, *dnmt1*, *bap1*, and *thoc1* may coordinately be syn-expressed, interact with each other, and function in the same processes during CNS development.

Cryptochromes (CRY) are involved in the circadian rhythms of plants and animals. In zebrafish, synchronization of circadian clock with cell cycle progression has been observed^52^. Compared to mammals, zebrafish retain six *cryptochrome* (*cry*) genes^53^. Also, it has been shown that Cry3a (also known as Cry1ba, a homolog for human CRY1) is able to negatively regulate transcriptional activity of circadian transcription factor Clock1a:Bmal1b^53^, indicating conserved functions for those clock genes among species. Expression of *cry3a* mRNA has been identified in ventral telencephalon, retina, and intestine at 5 dpf^32^. In adult zebrafish, it is expressed in the brain, muscle, heart, and the liver^31^. Although only weak protein interaction signals between Udu and Cry3a (Fig. 3) have been detected, the cellular function of circadian regulator CRY1 in mammals and the syn-expressing patterns of *cry3a* in zebrafish raise the necessity to investigate in the future whether Gon4l/Udu can influence cell cycle, DNA damage response, and genomic stability through interacting with Cry3a.

In addition to the genes discussed above, Cramped-like (Cramp1l or Crm) has been shown to interact with Gon4l in a large-scale human interactome study^21^. CRAMP1L and GON4L can also interact with MST1 when naïve CD4 + T cells differentiate into regulatory T cells (Treg) in another protein–protein interactome analysis^17^. Furthermore, Cramp1l is a polycomb group protein encoded gene that is dynamically regulated during cell cycle progression, peaks at S phase, and interacts with proliferating cell nuclear antigen (PCNA)^54,55^. In *cramp1l* mutants, the expression of histone H1, but not other histones, is greatly decreased^54^. Cramp1l has been shown to interact with another GON4L-interacting protein, NPAT (Nuclear Protein, Ataxia-Telangiectasia Locus)^16^. NPAT is a substrate for Cyclin E/CDK-2 and is responsible for biogenesis of histone locus body and histone gene transcription^15,56^. Therefore, Cramp1l and NPAT, together with Bap1, indicate that Gon4l/Udu may be essential for the connection between cell proliferation and histone expression/modification.

Gon4l has been considered as a negative transcription regulator that inhibits gene expression probably through interacting with YY1, SIN3A, and HDAC1. In this study, we have identified four novel Gon4l/Udu-interacting proteins. These newly identified proteins not only confirm the association of Gon4l/Udu with histone biogenesis, as demonstrated in previous reports, but also indicate that Gon4l/Udu can possibly regulate both histone modifications and CpG DNA methylation to influence various cellular events. Furthermore, Gon4l may also maintain genomic integrity while controlling transcription and cell proliferation. Overall, these findings provide a basis for better comprehending the pleiotropic effects of Gon4l/Udu.

## Methods

### Yeast two-hybrid (Y2H) screening

The yeast two-hybrid (Y2H) assays for zebrafish Udu and mouse Gon4l were performed by Hybrigenics (Paris, France) and Myriad Genetics (California, USA), respectively. Briefly, LexA and Gal4 system based on transcriptional activation of reporter genes was used to detect protein interactions. Bait fragments used for zebrafish Udu were the N-terminal CR1-CR3 (165–1,470 a.a.) and the C-terminal PAH and SANT domains (1,523–2,054 a.a.). The corresponding fragments of zebrafish Udu were cloned, checked by sequencing, and used as bait to screen for protein interactions using zebrafish embryo RP1 library (stages 18–20 hpf). A total of 56 million and 82 million colonies for Udu (165–1,470) and Udu (1,523–2,054) were analyzed for the interaction, respectively. The resulting sequences were searched against GenBank using an automated procedure. Bait fragments used for mouse Gon4l were the two PAH domains (1,458–1,857 a.a.) and SANT domain (1,858–2,261 a.a.). The two fragments were cloned and used for protein interactions with prey libraries from murine embryos, B lymphocytes (B cells), and cardiac myocytes. Around 5–10 million colonies were obtained after each mating, then picked from the selection plates and the prey inserts were identified by sequence analysis. To confirm the interactions, the bait and prey plasmid DNAs were isolated and co-transformed into a naïve yeast strain to recapitulate the interaction.

### Zebrafish maintenance and embryos

Zebrafish (*Danio rerio*) were raised and kept under standard conditions according to a previous report^57^. Wild-type and *udu*^*tu24*^ embryos were staged as described before^58^. All experimental procedures on zebrafish and their embryos were approved by the Institutional Animal Care and Use Committee of the National Health Research Institutes, Taiwan (NHRI-IACUC-106063-A) and carried out in accordance with the approved guidelines. Only zebrafish and no other animals were directly involved in this study.

### RNA Isolation and Reverse Transcription

RNA was isolated from WT embryos harvested at 1, 2, 3, 4, and 6 dpf, as well as from adult zebrafish livers, following the instructions of the NucleoSpin RNA kit (Macherey–Nagel GmbH & Co. KG). One microgram of total RNA was used for subsequent reverse transcription using GoScript Reverse Transcriptase (Promega). The obtained cDNA was used for subsequent PCR cloning.

### Plasmid construction

Full-length zebrafish *udu* C-terminally fused with Flag tag was PCR amplified. PCR product was subcloned into pcDNA3.1(+) at EcoRV/NotI sites. For the expression of PAH and SANT domains of Udu, pcDNA3.1(+)-flag-*udu*-P/S was used^19^. To perform co-IP analysis, candidate Udu-interacting genes were cloned from cDNA. For *bnipl*, *brd4*, *cry3a*, *ephx2*, *foxa2*, *gc*, *kif20a* and *lias*, sequence of Myc tag was added to their antisense primers. The PCR product of *cry3a* was digested with EcoRV/NotI and directly cloned into the corresponding sites of pcDNA3.1(+). The PCR products for *bnipl*, *brd4*, *ephx2*, *foxa2*, *gc*, *kif20a* and *lias* were first cloned into pJet1.2 vector and subcloned into pcDNA3.1(+) with NotI/XbaI. The PCR product for *bap1* was first cloned into pJet1.2 vector and subcloned into pCS2 + MT vector with XhoI/XbaI. The PCR products for *dnmt1* and *thoc1* were first cloned into pJet1.2 vector and subcloned into pcDNA3.1(+)-MT vector with EcoR1/Not1 and Not1/Xba1, respectively. The accession numbers for these genes and their corresponding primer pairs used in this study are listed in Supplementary Table S6. All PCR amplification of interested genes was performed using Q5 high fidelity polymerase PCR (NEB). Sequences for all the cloned genes were confirmed by the DNA Sequencing Core Lab (NHRI, Taiwan).

### Cell culture and transfection

HEK293 or COS-7 cells were cultured in Dulbecco’s modified Eagle’s medium (Invitrogen) supplemented with 10% fetal bovine serum and 1% penicillin/streptomycin (Invitrogen). For immunofluorescent staining, 2 × 10^6^ cells/well were seeded in a 6-well plate one day before transfection. One microgram of Myc-tagged constructs and/or one microgram Flag-tagged *udu* constructs were transfected into HEK293 cells using Lipofectamine 3,000 transfection reagent (Invitrogen). For co-IP analysis, 4 × 10^6^ HEK293 cells or 2 × 10^6^ COS-7 cells were seeded in a 60-mm dish one day before transfection. Two micrograms of Myc-tagged constructs and two micrograms Flag-tagged *udu* constructs were co-transfected. The pCS2-flag vector was used as a negative control for these experiments.

### Immunofluorescence staining and microscopic analysis

To examine the expression pattern of Udu, Udu-P/S and all the interacting candidate proteins, immunofluorescent staining was performed 1 day after transfecting plasmids into HEK293 cells. After washed with PBS, fixed with 4% paraformaldehyde and permeabilized with PBST (0.3% TritonX-100), the cells were blocked with 5% BSA/PBST. Incubation with mouse anti-FLAG antibody (F1804, 1:100 dilution; Sigma-Aldrich) or rabbit anti-MYC antibody (sc-789, 1:100 dilutions, Santa Cruz Biotechnology) at 4 °C overnight was followed by overnight incubation with Alexa Fluor 488-conjugated anti-mouse or Alexa Fluor 564-conjugated anti-rabbit secondary antibody (Molecular Probes). SlowFad Diamond Antifade Mountant with DAPI (Thermo Fisher Scientific) was used for nuclear counterstain and mounting. The fluorescent signals were taken by Axio Imager A1 (Zeiss) or TCS SP5 confocal microscope (Leica).

### Co-immunoprecipitation, SDS-PAGE and Western blot analysis

Two days after transfection, cells were harvested and lysed with Pierce IP Lysis Buffer (25 mM Tris–HCl pH 7.4, 150 mM NaCl, 1% NP-40, 1 mM EDTA, 5% glycerol) with the addition of protease inhibitor cocktail (Sigma, P8340). The lysates were clarified by centrifugation and determined for their concentration using Rapid Gold BCA Protein Assay Kit (Thermo Fisher Scientific). A total of 40 μl of anti-FLAG M2 magnetic beads (Sigma-Aldrich) was washed with lysis buffer and added to 800 μg of total protein lysates. After overnight incubation at 4 °C, supernatants were removed and the magnetic beads were washed in lysis buffer for 4 times. A total of 80 μl of co-IP elution buffer (0.1 M glycine HCl, pH 3.5, buffer) was added to each sample. The samples were incubated with gentle shaking for 5 min at room temperature to elute precipitated proteins. Then the magnetic beads were spun down and the supernatants were transferred immediately to fresh Eppendorf vials containing 10 μl of neutralizing buffer (0.5 M Tris–HCl, pH 7.4, 1.5 M NaCl). SDS-PAGE and subsequent Western blot analyses were performed with mouse anti-FLAG M2 (F1804, 1:5,000 dilution, Sigma-Aldrich) or rabbit anti-MYC antibody (sc-789, 1:10,000 dilution, Santa Cruz Biotechnology). To enhance the signals, Western Blot Ag Signal Enhancer (Biomate) was used prior blocking the PVDF membranes with 5% skim milk (BD Difco) according to the manufacturer’s instructions. Mouse or rabbit secondary antibody conjugated with HRP (1:10,000 dilution; R&D Systems) was applied. The chemiluminescent signals were visualized by Immobilon HRP substrate kit (Millipore) and captured by the BioSpectrumAC Imaging System (UVP). Full-length blots are included in Supplementary Fig. S7 as supporting information. Western blots for Bap1, Dnmt1, and Thoc 1 were run in the same gel, while Cry3a was run separately.

### Phenotypic enrichment analysis using model organism phenotype enrichment analysis (modPhEA)

The model organism Phenotype Enrichment Analysis (modPhEA) is a freely available tool that collects phenotypic data from both mutagenesis and knockdown animal experiments among different species, including budding yeast, roundworm, fruit fly, zebrafish, mouse, and human^28^. modPhEA reports enriched phenotypes that are associated with a given group of genes. Therefore, modPhEA was used to analyze phenotypes enriched by Gon4l/Udu-interacting proteins. Twenty genes encoding for Gon4l/Udu-interacting proteins with mouse Ensembl IDs were input manually as dataset 1 and compared against the rest of the genes in the genome that represent the background (dataset 2). Enrichment analyses were performed based on phenotype databases from either mouse or *Caenorhabditis elegans* because of their most abundant results obtained after analyses. To calculate and compare the differences between these two groups of genes (set 1 and set 2), Fisher’s Exact Test with differentially enriched hypothesis was applied. The Benjamini–Hochberg Procedure was used for correction of the p-value to decrease the false-discovery rate.

### Probe preparation and in situ hybridization

For *bap1* probe template preparation, an 842 bp fragment of *bap1* C-terminal was PCR amplified from diluted pCS2-MT-*bap1* plasmid DNA (primer pair was listed in Supplementary Table S6). The PCR product was cleaned up, and ligated to pJet1.2 vector. After checking the orientation of the *bap1* insert, the pJet1.2-*bap1*-antisense-842 bp plasmid was linearized with Xba1 for subsequent probe synthesis using MEGAscript T7 Transcription kit (Thermo Fisher Scientific). For *dnmt1* probe synthesis, a 3,197 bp fragment of *dnmt1* was PCR-amplified from pcDNA3-MT-*dnmt1* plasmid DNA (primer pair was listed in Supplementary Table S6). The PCR product was cleaned up, and ligated to pJet1.2 vector. After checking the orientation of the *dnmt1* insert, the plasmid was linearized with EcoR1 for subsequent probe synthesis with T7 polymerase (Thermo Fisher Scientific). For *thoc1* probe synthesis, we cut the ready-made pJet1.2-*thoc1*-antisense full-length plasmid with SacI. After enzyme digestion, a 649 nt *thoc1* probe was synthesized by MEGAscript T7 Transcription kit. For *cry3a* probe synthesis, a 1865 bp fragment of *cry3a* was PCR-amplified from pcDNA3-*cry3a*-MT plasmid DNA (primer pair was listed in Supplementary Table S6). The PCR product was cleaned up, and ligated to pJet1.2 vector. After checking the orientation, the plasmid was linearized with Xba1 for subsequent probe synthesis with T7 polymerase (Thermo Fisher Scientific). For *udu* probe synthesis, we cloned full-length *udu* into pGEMT-easy. After checking the orientation of the insert, the plasmid was digested with BamHI and a probe around 4.1 kb was synthesized using MEGAscript SP6 Transcription kit (Thermo Fisher Scientific). The *bap1* and *thoc1* probes were labeled with fluorescein labeling mix (Roche), while the *udu* probe was labeled with digoxigenin labeling mix (Roche). Detailed methods for probe synthesis and removal of template DNA were according to the manufacturer’s protocol. To examine the expression patterns of *bap1*, *thoc1*, and *udu* during zebrafish development, embryos/larvae were harvested at 3, 6, 22, 24, 36, 48, and 72 hpf. Whole mount in situ hybridization was performed as described previously^59^. Embryos were mounted in glycerol (Sigma) or Murray’s Clear (BABB, benzyl alcohol: benzyl benzoate = 1:2), and images were taken by Zeiss Axiovision Imager A1 or Zeiss Discovery V8.

### Quantitative real time PCR (qPCR) analysis

RNA was extracted from wild type and *udu*^*tu24*^ embryos at 22 hpf as previously described. 1 μg of total RNA was reversely transcribed, and the cDNA was diluted and amplified with the respective primers listed in Supplementary Table S6. *gapdh* was used as an internal control. The SYBR Green-based qPCR was carried out on Roche LC480 II using SensiFAST SYBR Hi-ROX Kit (Bioline). The program for qPCR was as follows: one cycle of 95 °C for 5 min, followed by 45 cycles of 95 °C for 10 s, 60 °C for 10 s, and 72 °C for 20 s. A program for melting curve was added to examine for the existence of primer dimers or non-specific signals. Data were expressed as fold-change compared to wild type sample (mean ± SD).

## Supplementary information

Supplementary file1
