# Supplementary material for: Newly identified Gon4l/Udu-interacting proteins implicate novel functions
Source: Sci Rep. 2020 Aug 26;10:14213. doi: 10.1038/s41598-020-70855-9 (PMC7449961; doi:10.1038/s41598-020-70855-9)
Supplement: Supplementary file 1 — Supplementary file1 [file 41598_2020_70855_MOESM1_ESM.pdf]

Supplementary Information

for

Newly identified Gon4l/Udu-interacting proteins implicate novel functions

Su-Mei Tsai[1], Kuo-Chang Chu[1] & Yun-Jin Jiang[1,2,3,4,5*]

[1]Institute of Molecular and Genomic Medicine, National Health Research Institutes, Zhunan, Miaoli County, Taiwan

[2]Laboratory of Developmental Signalling and Patterning, Institute of Molecular and Cell Biology, Singapore

[3]Biotechnology Center, National Chung Hsing University, Taichung, Taiwan

[4]Institute of Molecular and Cellular Biology, National Taiwan University, Taipei, Taiwan

[5]Department of Life Science, Tunghai University, Taichung, Taiwan

Supplementary Table S1. Physically Gon4l/Udu-interacting proteins of humans collected from BioGRID database.

Official Symbol Interactor	Synonyms of Interactor	Experimental System	GO terms	Reference (PMID)
CRAMP1L	HN1L|TCE4	Affinity Capture-MS	chromatin binding; DNA-binding transcription factor activity, RNA polymerase II-specific	28514442 30413360
H2AFX	H2A.X|H2A/X|H2AX	Affinity Capture-MS	damaged DNA binding; DNA binding; histone binding; protein heterodimerization activity	28514442 28242625
HIST1H2AB	H2A/m|H2AFM	Affinity Capture-MS	DNA binding; protein heterodimerization activity	27248496
HIST1H2BA	H2BFU|STBP|TSH2B|TSH2B.1|bA317E16.3	Affinity Capture-MS	DNA binding; histone binding; protein heterodimerization activity	28514442 26186194
HIST1H2BB	H2B.1|H2B/f|H2BFF	Affinity Capture-MS	DNA binding; histone binding; protein heterodimerization activity	27248496
HIST1H2BG	H2B.1A|H2B/a|H2BFA|dJ221C16.8	Proximity Label-MS	DNA binding; histone binding; protein heterodimerization activity	25281560
ESR2	ER-BETA|ESR-ETA|ESRB|ESTRB|Erb|NR3A2	Affinity Capture-MS	DNA-binding transcription factor activity, RNA polymerase II-specific; estrogen receptor activity; nuclear receptor activity	29509190
DHX16	DBP2|DDX16|PRO2014|PRP8|PRPF2|Prp2	Co-fractionation	ATPase activity; RNA helicase activity; mRNA splicing	22939629
PNKD	BRP17|DYT8|FPD1|KIPP1184|MR-1|MR1|PDC|PKND1|TAHCCP2	Affinity Capture-MS	hydroxyacylglutathione hydrolase activity; metal ion binding; regulation of synaptic transmission, dopaminergic	28514442 26186194
ASB3	ASB-3	Affinity Capture-MS	post-translational protein modification; protein ubiquitination	28514442

Supplementary Table S2. List of Gon4l/Udu-interacting proteins used for model organism Phenotype Enrichment Analysis.

Symbol	Name	Ensembl no. (mouse homolog)	Reference (PMID)/Source	phenotype annotated
MCM3	minichromosome maintenance complex component 3, mRNA	ENSMUSG00000041859	19656853 and this study	y
MCM4	minichromosome maintenance complex component 4 , mRNA	ENSMUSG00000022673	19656853 and this study	y
NPAT	Nuclear Protein, Ataxia-Telangiectasia Locus	ENSMUSG00000033054	25339177 and this study	y
CRY1	cryptochrome circadian regulator 1ba, transcript variant 1, mRNA	ENSMUSG00000020038	this study	y
THOC1	Tho complex 1, also known as Tho1, HPR1	ENSMUSG00000024287	this study	y
BAP1	BRCA1-associated protein 1	ENSMUSG00000021901	this study	y
DNMT1	DNA methyltransferase 1	ENSMUSG00000004099	this study	y
CRAMP1L	Cramped-like, cramp1L	ENSMUSG00000038002	BioGRID[3.5] and this study	y
H2AX	H2A histone family member X	ENSMUSG00000049932	BioGRID[3.5]	y
HIST1H2AB	Histone cluster 1 H2A family member b	ENSMUSG00000061615	BioGRID[3.5]	n
HIST1H2BA	Histone cluster 1 H2B family member a	ENSMUSG00000050799	BioGRID[3.5]	n
HIST1H2BB	Histone cluster 1 H2B family member b	ENSMUSG00000075031	BioGRID[3.5]	n
HIST1H2BG	Histone cluster 1 H2B family member g	ENSMUSG00000058385	BioGRID[3.5]	n
ESR2	Estrogen receptor 2	ENSMUSG00000021055	BioGRID[3.5]	y
ASB3	Ankyrin repeat and SOCS box containing 3	ENSMUSG00000020305	BioGRID[3.5]	n
PNKD	PNKD metallo-beta-lactamase domain containing	ENSMUSG00000026179	BioGRID[3.5]	y
DHX16	DEAH (Asp-Glu-Ala-His) box polypeptide 16	ENSMUSG00000024422	BioGRID[3.5]	y
YY1	Ying-Yang 1 transcription factor	ENSMUSG00000021264	21454521 and BioGRID[3.5]	y
SIN3A	SIN3 transcription regulator family member A	ENSMUSG00000042557	21454521 and BioGRID[3.5]	y
HDAC1	Histone deacetylase 1	ENSMUSG00000028800	21454521 and BioGRID[3.5]	y

Supplementary Table S3: Gon4L/Udu-interacting protein-enriched phenotypes of mouse phenome database (Gene Expression Database (GXD), Mouse Genome Informatics Web Site, MGI6.14).

Phenotype Name	Phenotype ID	BH FDR corrected P-value	Genes with the term
increased thymus tumor incidence	MP:0010365	0.006	mcm4, h2afx
increased T cell derived lymphoma incidence	MP:0002024	0.006	mcm4, h2afx
embryonic lethality, complete penetrance	MP:0011092	0.007	mcm4, dnmt1, yy1, hdac1
increased hemolymphoid system tumor incidence	MP:0010296	0.007	mcm4, esr2, h2afx
prenatal lethality	MP:0002080	0.012	mcm3, mcm4, thoc1, bap1, dnmt1, yy1, sin3a, hdac1
decreased cell proliferation	MP:0000352	0.012	mcm3, mcm4, dnmt1, h2afx, hdac1
abnormal dopamine level	MP:0001905	0.014	esr2, pnkd
embryonic lethality	MP:0008762	0.014	mcm4, thoc1, bap1, dnmt1, yy1, sin3a, hdac1
increased organ/body region tumor incidence	MP:0010274	0.014	mcm4, esr2, h2afx
increased classified tumor incidence	MP:0010273	0.014	mcm4, esr2, h2afx
abnormal classified tumor incidence	MP:0020188	0.016	mcm4, esr2, h2afx
abnormal organ/body region tumor incidence	MP:0013152	0.016	mcm4, esr2, h2afx
chromosomal instability	MP:0008866	0.018	mcm4, h2afx
abnormal apoptosis	MP:0001648	0.021	mcm3, thoc1, dnmt1, esr2, h2afx
abnormal cell proliferation	MP:0000350	0.022	mcm3, mcm4, dnmt1, esr2, h2afx, hdac1
embryo phenotype	MP:0005380	0.023	mcm4, npat, thoc1, bap1, dnmt1, yy1, hdac1
increased cell death	MP:0012556	0.024	mcm3, thoc1, dnmt1, esr2
increased tumor incidence	MP:0002020	0.024	mcm4, esr2, h2afx
increased apoptosis	MP:0006042	0.024	mcm3, thoc1, dnmt1, esr2
increased embryonic tissue cell apoptosis	MP:0013504	0.028	thoc1, dnmt1
increased lymphoma incidence	MP:0012431	0.028	mcm4, h2afx

Supplementary Table S3: continued

Phenotype Name	Phenotype ID	BH FDR corrected P-value	Genes with the term
embryonic lethality prior to organogenesis	MP:0013292	0.029	mcm4, thoc1, bap1, sin3a
embryonic lethality between implantation and placentation	MP:0009850	0.029	mcm4, bap1, sin3a
abnormal cell cycle	MP:0003077	0.03	mcm3, mcm4, hdac1
increased gland tumor incidence	MP:0010288	0.033	mcm4, h2afx
abnormal apocrine gland morphology	MP:0013554	0.034	mcm4, esr2
embryonic lethality between implantation and somite formation, complete penetrance	MP:0011096	0.036	mcm4, sin3a
abnormal chromosome stability	MP:0010094	0.036	mcm4, h2afx
abnormal embryonic tissue cell apoptosis	MP:0013503	0.04	thoc1, dnmt1
abnormal mammary gland morphology	MP:0000627	0.04	mcm4, esr2
preweaning lethality, complete penetrance	MP:0011100	0.04	mcm4, npat, bap1, dnmt1, hdac1
embryonic lethality prior to tooth bud stage	MP:0013293	0.046	mcm4, thoc1, bap1, sin3a

Supplementary Table S4: Bap1-, Dnmt1-, and Thoc1-enriched phenotypes of mouse phenome database (Gene Expression Database (GXD), Mouse Genome Informatics Web Site, MGI6.14).

Phenotype Name	Phenotype ID	BH FDR corrected P-value	Genes with the term (Ensembl no.)
increased embryonic tissue cell apoptosis	MP:0013504	0.001	thoc1, dnmt1
abnormal embryonic tissue cell apoptosis	MP:0013503	0.003	thoc1, dnmt1
abnormal embryo development	MP:0001672	0.006	thoc1, bap1, dnmt1
abnormal embryonic tissue physiology	MP:0008932	0.006	thoc1, dnmt1
embryonic lethality	MP:0008762	0.012	thoc1, bap1, dnmt1
increased apoptosis	MP:0006042	0.015	thoc1, dnmt1
embryonic lethality prior to organogenesis	MP:0013292	0.017	thoc1, bap1
prenatal lethality	MP:0002080	0.021	thoc1, bap1, dnmt1
increased cell death	MP:0012556	0.021	thoc1, dnmt1
embryonic lethality prior to tooth bud stage	MP:0013293	0.021	thoc1, bap1
embryo phenotype	MP:0005380	0.024	thoc1, bap1, dnmt1
abnormal embryonic growth/weight/body size	MP:0002088	0.033	thoc1, bap1
abnormal apoptosis	MP:0001648	0.035	thoc1, dnmt1
preweaning lethality, complete penetrance	MP:0011100	0.044	dnmt1, bap1
abnormal cell death	MP:0000313	0.046	thoc1, dnmt1

Supplementary Table S5: Gon4l/Udu-interacting protein-enriched phenotypes of *Caenorhabditis elegans* database from modPhEA (WBPhenotype, data version: WormBase web site, http://www.wormbase.org, release WS273, November 2019).

Phenotype Name	Phenotype ID	BH FDR corrected P-value	Genes with the term (Ensembl no.)
nuclear morphology variation early emb	WBPhenotype:0001026	0.00001194	mcm4, h2afx, hist1h2ab
nuclear appearance number defective early emb	WBPhenotype:0001035	0.00002388	mcm3, mcm4, h2afx, hist1h2ab
sister chromatid segregation defective early emb	WBPhenotype:0000772	0.00006323	mcm3, h2afx, hist1h2ab, hist1h2ba
nucleus defective early emb	WBPhenotype:0001138	0.0001597	mcm3, mcm4, h2afx, hist1h2ab
embryonic cell morphology variant	WBPhenotype:0000770	0.0003568	mcm3, mcm4, h2afx, hist1h2ab
chromosome segregation variant	WBPhenotype:0000773	0.0003568	mcm3, h2afx, hist1h2ab, hist1h2ba, hdac1
pharyngeal development variant	WBPhenotype:0000707	0.0003568	h2afx, hist1h2ab, hist1h2ba, hdac1
pronuclear nuclear appearance variant emb	WBPhenotype:0001374	0.0006685	mcm3, mcm4, h2afx, hist1h2ab
protruding vulva	WBPhenotype:0000697	0.0006685	mcm3, h2afx, hist1h2ab, hist1h2ba, sin3a, hdac1
pronuclear nuclear appearance defective early emb	WBPhenotype:0001034	0.0006685	mcm3, mcm4, h2afx, hist1h2ab
cell corpse degradation variant	WBPhenotype:0010004	0.0006685	h2afx, hist1h2ab, hist1h2ba
anaphase bridging	WBPhenotype:0001875	0.0006685	h2afx, hist1h2ab, hist1h2ba
vulva morphology variant	WBPhenotype:0000695	0.0009539	mcm3, h2afx, hist1h2ab, hist1h2ba, sin3a, hdac1
hermaphrodite reproductive system morphology variant	WBPhenotype:0001335	0.0009645	mcm3, h2afx, hist1h2ab, hist1h2ba, sin3a, hdac1
cytokinesis defective early emb	WBPhenotype:0001078	0.0009736	mcm3, h2afx, hist1h2ab, hist1h2ba
cytokinesis variant emb	WBPhenotype:0001018	0.001	mcm3, h2afx, hist1h2ab, hist1h2ba
mitotic chromosome segregation variant	WBPhenotype:0001378	0.001	h2afx, hist1h2ab, hist1h2ba
body elongation defective	WBPhenotype:0000242	0.001	h2afx, hist1h2ab, hist1h2ba
early embryonic lethal	WBPhenotype:0001100	0.001	mcm3, mcm4, h2afx, hist1h2ab, hist1h2ba, hdac1

Supplementary Table S5: continued

Phenotype Name	Phenotype ID	BH FDR corrected P-value	Genes with the term (Ensembl no.)
body elongation variant	WBPhenotype:0001478	0.002	h2afx, hist1h2ab, hist1h2ba
organ system development variant	WBPhenotype:0000530	0.003	mcm3, mcm4, h2afx, hist1h2ab, hist1h2ba, sin3a, hdac1
embryonic cell organization biogenesis variant	WBPhenotype:0000764	0.003	mcm3, mcm4, h2afx, hist1h2ab
cytokinesis variant	WBPhenotype:0002408	0.003	mcm3, h2afx, hist1h2ab, hist1h2ba
programmed cell death variant	WBPhenotype:0001172	0.004	mcm4, h2afx, hist1h2ab, hist1h2ba, hdac1
embryonic arrest	WBPhenotype:0000867	0.004	h2afx, hist1h2ab, hist1h2ba, hdac1
apoptosis variant	WBPhenotype:0000730	0.004	mcm4, h2afx, hist1h2ab, hist1h2ba, hdac1
thin	WBPhenotype:0000164	0.004	mcm4, h2afx, hist1h2ab, hist1h2ba
pattern of transgene expression variant	WBPhenotype:0000961	0.004	mcm3, mcm4, h2afx, hist1h2ab, hist1h2ba, sin3a, hdac1
reproductive system morphology variant	WBPhenotype:0000605	0.007	mcm3, h2afx, hist1h2ab, hist1h2ba, sin3a, hdac1
embryonic cell physiology variant	WBPhenotype:0000763	0.007	mcm3, mcm4, h2afx, hist1h2ab
cell death variant	WBPhenotype:0000729	0.007	mcm4, h2afx, hist1h2ab, hist1h2ba, hdac1
alimentary system development variant	WBPhenotype:0000617	0.007	h2afx, hist1h2ab, hist1h2ba, hdac1
organism hypertonic lethality increased	WBPhenotype:0001751	0.008	h2afx, hist1h2ab
transgene expression increased	WBPhenotype:0001236	0.008	h2afx, hist1h2ab, hist1h2ba, sin3a, hdac1
maternal sterile	WBPhenotype:0000689	0.008	mcm3, h2afx, hist1h2ab, hist1h2ba, hdac1
chromosome condensation variant	WBPhenotype:0001361	0.009	h2afx, hist1h2ab, hist1h2ba
stress induced lethality increased	WBPhenotype:0000141	0.009	h2afx, hist1h2ab
hermaphrodite sterile	WBPhenotype:0000694	0.009	mcm3, h2afx, hist1h2ab, hist1h2ba, hdac1

Supplementary Table S6. Primers used in this study.

Gene Name	NCBI Accession No.	Forward primer	Reverse primer	Application
udu	NM_001201535.1	gatccaccatgggatggaaacgcaagtc	aaggcggccgctcacttgtcgtcatcgtctttgtagtcgtcctgctcttcatcag	Cloning-expression
bap1	NM_001163837.1	ctcgagatgaacaaaggttggctgg	tctagactattgccgtttggcttt	Cloning-expression
		gtcacaaacagcagggttcg	gtcctcctctggtcatcaatctt	Cloning-probe
bnipl	XM_009292526.3	tttccaccatggggacttactcagac	aactcacagatcctcttctgagatgagtttttgttccctgttcagctgcgtatc	Cloning-expression
brd4	NM_001111281.1	ccgccaccatgggggacggcctggac	gccttacagatcctcttctgagatgagtttttgttcgaacaagttctcctcaaa	Cloning-expression
cry3a	NM_001313822.1	cagccaccatggccccaaattccatc	tgtgcggccgcttacagatcctcttctgagatgagttttgttcactcctcatacttgactg	Cloning-expression
dnmt1	NM_131189.2	cgagaattcaatgcctaccaagacctca	gaagcggccgcttagtcagagagctcca	Cloning-expression
ephx2	XM_021467153.1	agtccaccatgaagaaggcagtgttg	ggttcacagatcctcttctgagatgagtttttgttctaacttgggatgaacaga	Cloning-expression
foxa2	NM_130949.2	tttccaccatgctcggtgctgtcaaa	aaattacagatcctcttctgagatgagtttttgttcggaagagttcaggatggg	Cloning-expression
gc	NM_001002568.2	aatccaccatgatgaatgcatcttta	tggttacagatcctcttctgagatgagtttttgttcagcagcactgtcatcatc	Cloning-expression
kif20a	NM_001161470.1	ctgccaccatggctctggcgtctcca	aaattacagatcctcttctgagatgagtttttgttcatacttgactcggagcat	Cloning-expression
lias	NM_001110401.1	ctaccaccatggcgttgatcagtaga	agtttacagatcctcttctgagatgagtttttgttcctctgctgttgctgtggt	Cloning-expression
thoc1	NM_201324.1	tgcgcggccgcatgtctcctccgtctcac	tcgtctagattatgtttcgctcagatt	Cloning-expression
cebpa	NM_131885.2	catctacgacagccaagcaa	ctggagatgcatggtggttt	qPCR
gapdh	NM_001115114.1	gtggagtctactggtgtcttc	gtgcaggaggcattgcttaca	qPCR

Supplementary Figures

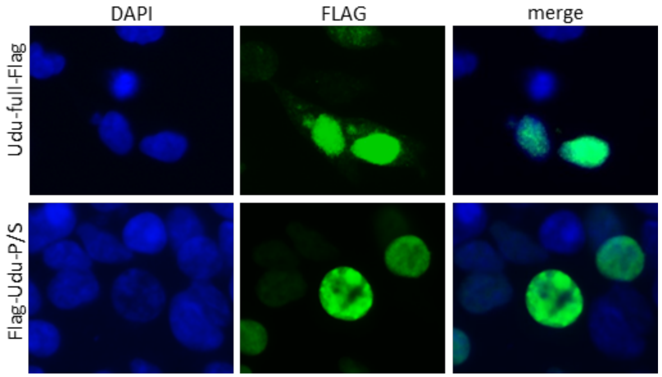

Supplementary Figure S1. Immunofluorescent staining for the nuclear localization of Udu.
HEK293 cells were transfected with either Flag-tagged Udu-full (upper panels) or Udu-P/S (lower panels). For subsequent immunofluorescent staining analysis, anti-FLAG M2 monoclonal antibody and Alexa Fluor 488-conjugated anti-mouse secondary antibody were used. Images were taken under fluorescent microscope. Green fluorescence indicates Udu-full or Udu-P/S. Blue color is DAPI used for nuclear counterstain.

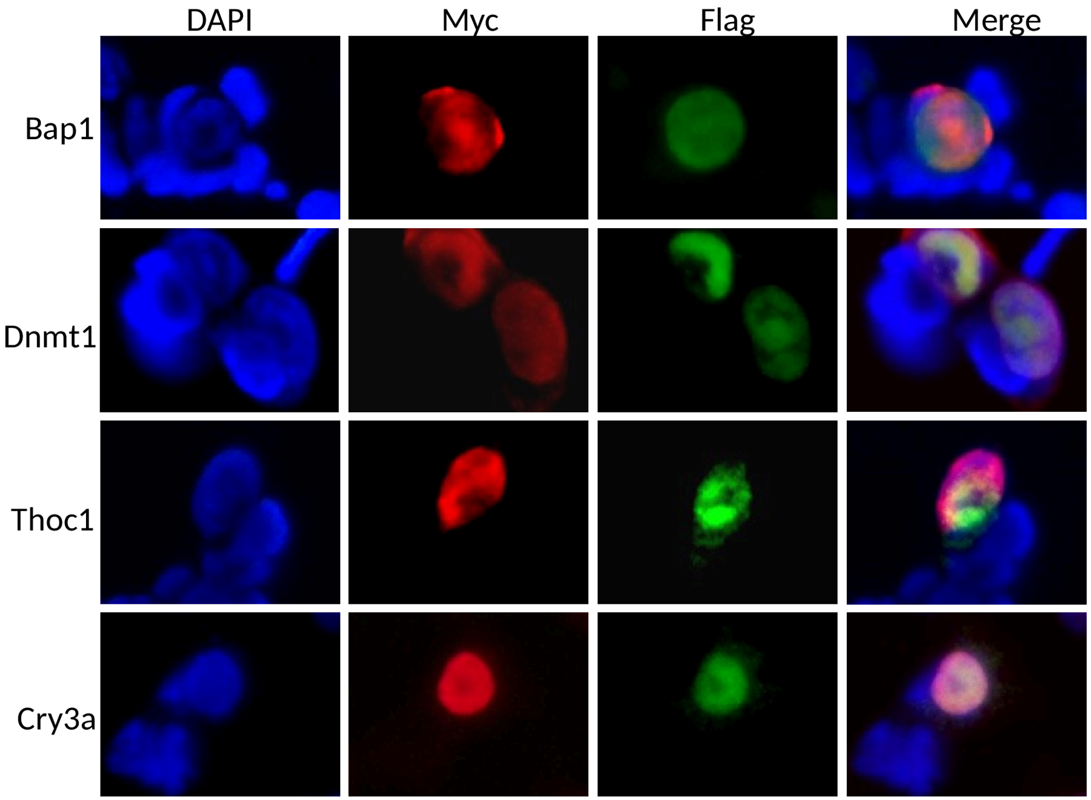

Supplementary Figure S2. Immunofluorescent staining for the cellular co-localization analysis of Udu and its interacting proteins. Flag-tagged Udu-full construct was co-transfected individually with each Myc-tagged construct, including Bap1, Dnmt1, Thoc1, and Cry3a. Anti-FLAG M2 monoclonal antibody and rabbit anti-MYC antibody were used. Alexa Fluor 488-conjugated anti-mouse secondary antibody and Alexa Fluor 564-conjugated anti-rabbit secondary antibody were then applied. Green fluorescence indicates Udu-full, while red fluorescence indicates the expression of Myc-tagged proteins. Blue color is DAPI used for nuclear counterstain. Merged images show the nuclear co-localization of Udu and the interacting proteins.

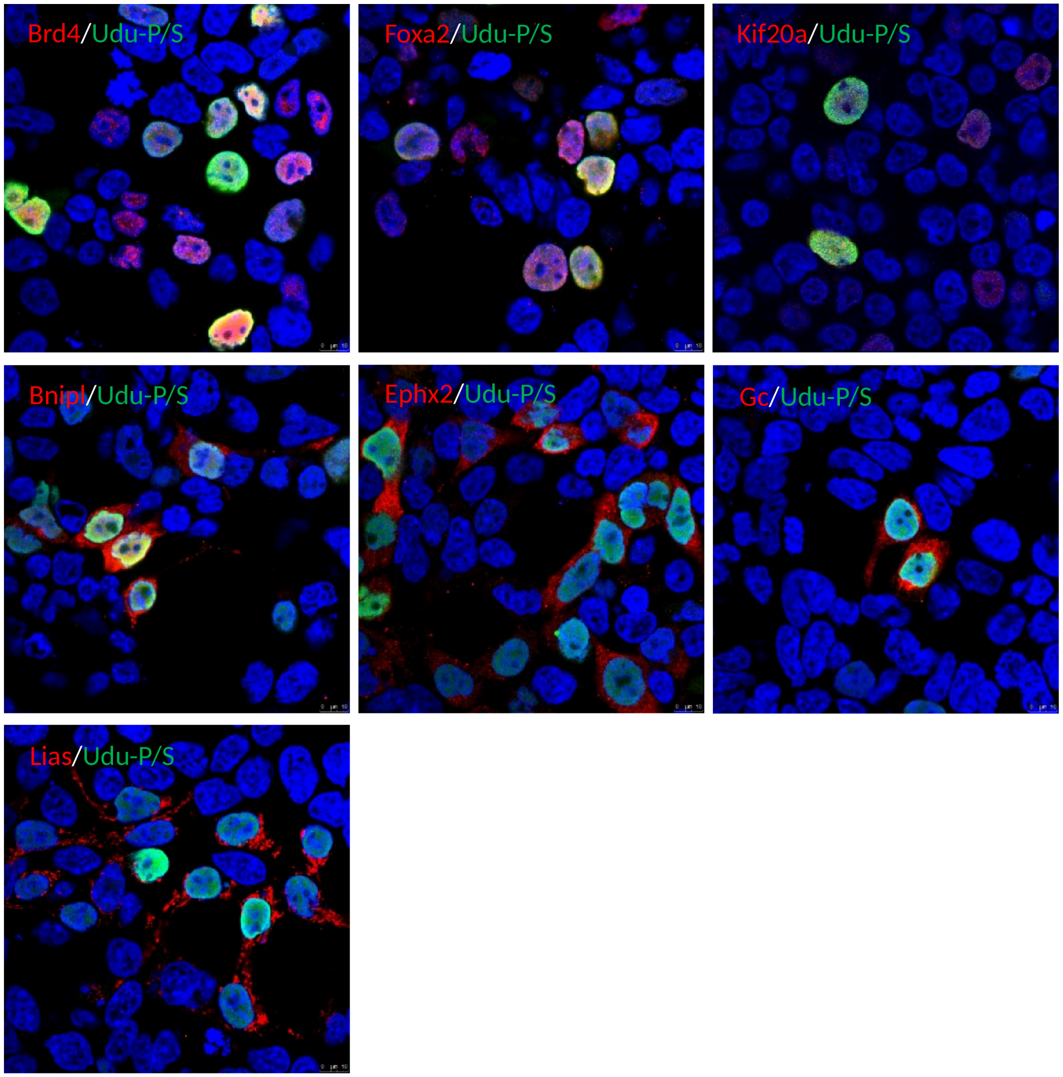

Supplementary Figure S3. Confocal images for the cellular co-localization analysis of Udu and its candidate interacting proteins. For co-localization analysis, Flag-tagged Udu-P/S construct was co-transfected individually with each Myc-tagged construct, including the nucleus-localized Brd4, Foxa2, Kif20a, and Bnipl, the cytoplasm-localized Bnipl, Ephx2 and Gc, and the mitochondrial protein Lias (**Ref.**). Note that Bnipl is expressed both in the nucleus and in the cytoplasm. Anti-FLAG M2 monoclonal antibody and rabbit anti-MYC antibody were used. Alexa Fluor 488-conjugated anti-mouse secondary antibody and Alexa Fluor 564-conjugated anti-rabbit secondary antibody were then applied. Green fluorescence indicates Udu-P/S, while red fluorescence indicates the expression of Myc-tagged proteins. Blue color is DAPI used for nuclear counterstain.

Ref.: Mayr, J.A., Zimmermann, F.A., Fauth, C., Bergheim, C., Meierhofer, D., Radmayr, D., Zschocke, J., Koch, J., Sperl, W., 2011. Lipoic acid synthetase deficiency causes neonatal-onset epilepsy, defective mitochondrial energy metabolism, and glycine elevation. *Am. J. Hum. Genet.* **89**, 792-797.

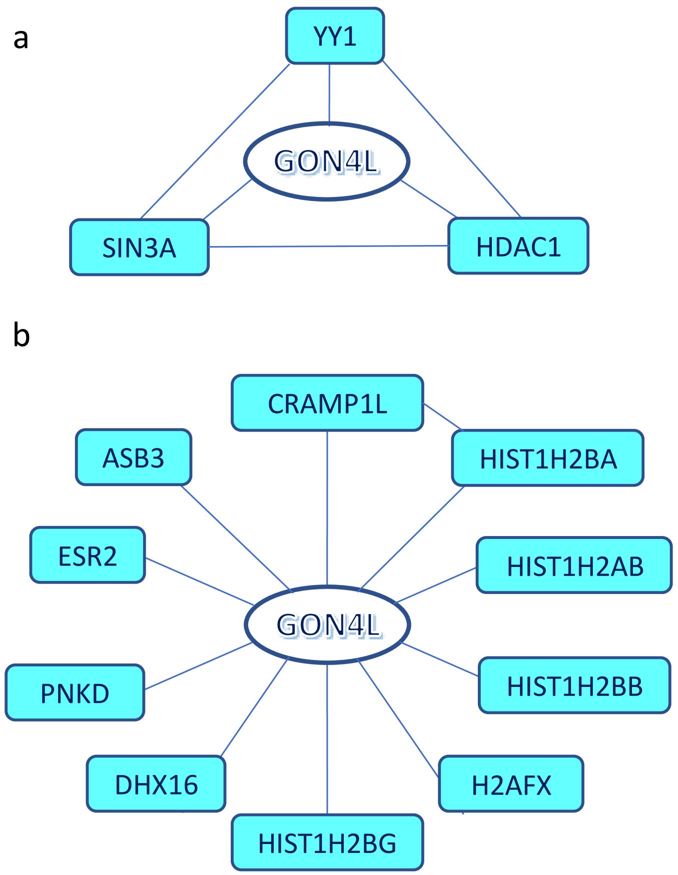

Supplementary Figure S4. Networks between Gon4l and its interactors.

Physical interactors for Gon4l were identified from (a) mouse or (b) human datasets of BioGRID.

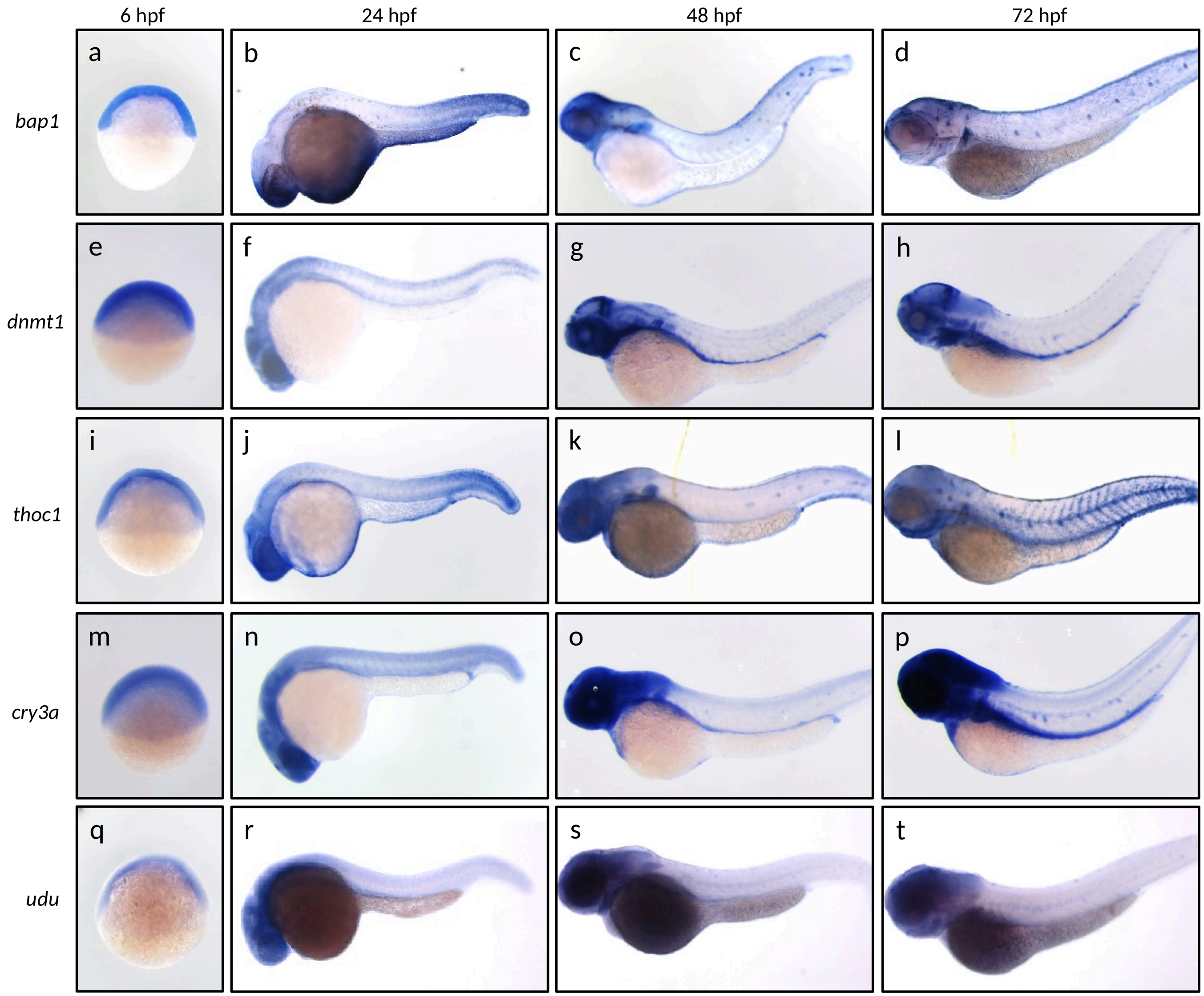

Supplementary Figure S5. Whole mount *in situ* hybridization analysis for the expression of *bap1*, *dnmt1*, *thoc1*, *cry3a*, and *udu* genes during zebrafish early development. Wild type embryos were harvested to analyze the temporal and spatial expressions of (a–d) *bap1*, (e–h) *dnmt1*, (i–l) *thoc1*, (m–p) *cry3a*, and (q–t) *udu* genes at indicated time points. Embryos are orientated with anterior to the left.

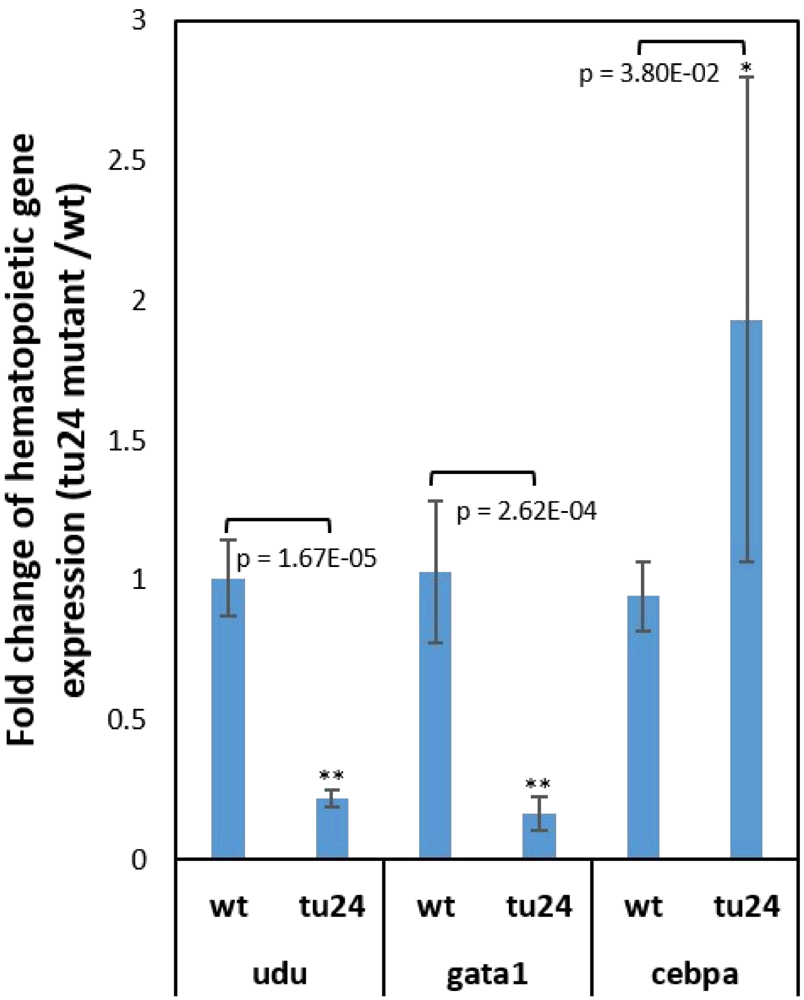

Supplementary Figure S6. Quantitative real-time PCR (Q-PCR) analysis for *cebpa* expression.

Expression levels of *udu*, *gata1* and *cebpa* in *udu*^{tu24} mutants at 22 hpf were detected and compared with that in WT larvae at the same stage by relative Q-PCR analysis. Data were normalized to *gapdh* and expressed as fold-induction compared to WT (mean +/- SD). *, $p<0.05$; **, $p<0.001$.

a

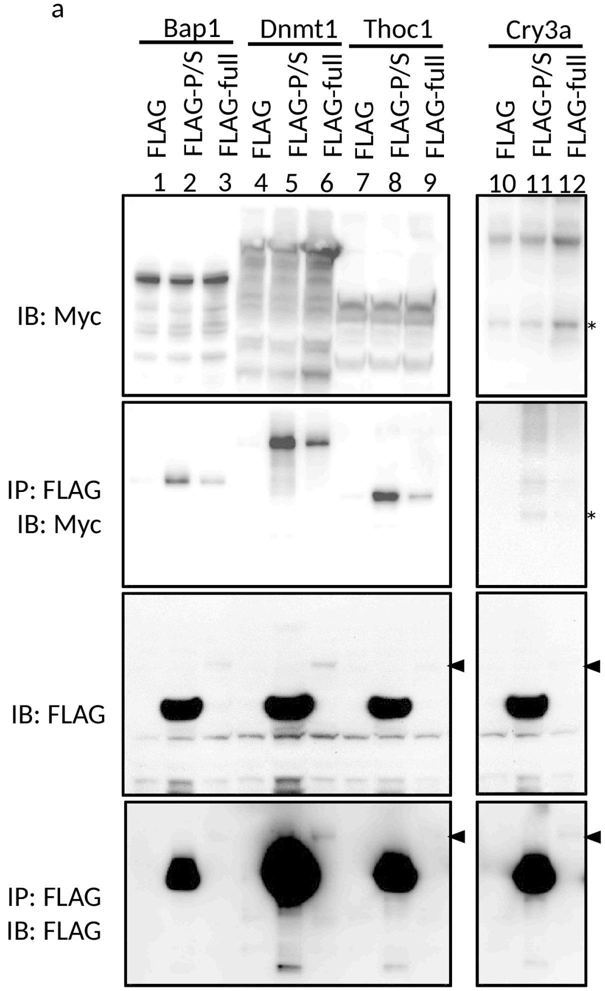

b

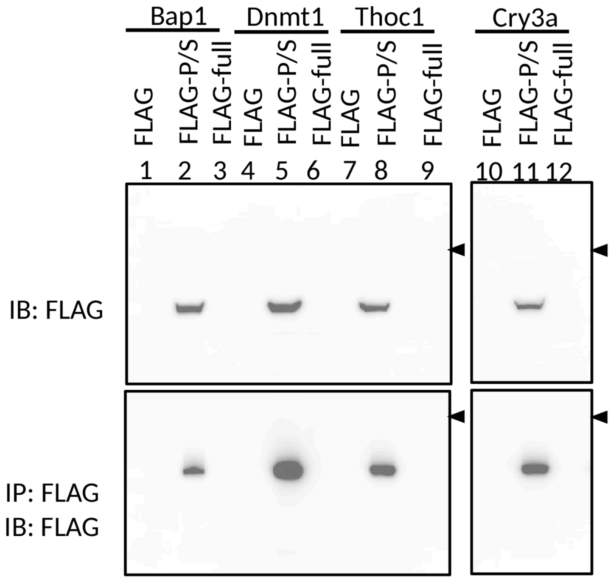

Supplementary Figure S7. Co-immunoprecipitation and Western blot analysis for the interaction of Udu with Bap1, Dnmt1, Thoc1, and Cry3a.
(a) Full-length blots of Figure 3 are shown here as supporting information. Samples for Bap1, Dnmt1 and Thoc1 were loaded in the same gel, while Cry3a was loaded in the other. To observe the weak expression of full-length Udu, anti-FLAG immunoblot was detected for longer exposure time as shown in panel a. The asterisk indicates the band for Cry3a. Arrowheads indicate the expected size for full-length Udu. (b) Shorter exposure time for FLAG Western blots are also shown.
